# *Tenebrio molitor* Meal-Induced Changes in Rat Gut Microbiota: Microbiological and Metagenomic Findings

**DOI:** 10.3390/ijms26178663

**Published:** 2025-09-05

**Authors:** Remigiusz Gałęcki, Adriana Nowak, Justyna Szulc

**Affiliations:** 1 Department of Veterinary Prevention and Feed Hygiene, Faculty of Veterinary Medicine, University of Warmia and Mazury in Olsztyn, Oczapowskiego 13, 10-719 Olsztyn, Poland; 2 Department of Environmental Biotechnology, Faculty of Biotechnology and Food Sciences, Lodz University of Technology, Wólczańska 171/173, 90-530 Lodz, Poland

**Keywords:** edible insects, model organisms, metagenome analysis, microbiome, nutrition, yellow mealworm, novel food, laboratory rats

## Abstract

As demand for sustainable protein sources grows, edible insects like *Tenebrio molitor* (yellow mealworm) are gaining attention as functional feed ingredients. This study investigated how dietary inclusion of *T. molitor* meal affects gut microbiota composition and diversity in laboratory rats. Wistar rats were divided into three diet groups: standard feed, 35% chicken meal, and 35% *T. molitor* meal. Fecal samples were collected at weeks 4, 6, and 8. Microbial populations were assessed using culture-based methods, and community structure was analyzed at week 9 via Illumina MiSeq 16S rRNA sequencing. Bioinformatic analyses evaluated microbial diversity and predicted functions. Rats fed *T. molitor* meal showed significantly reduced counts of total aerobic/anaerobic bacteria, fungi, and coagulase-positive staphylococci. Metagenomics revealed a Firmicutes-dominated microbiota, with enrichment of protein- and cholesterol-metabolizing taxa (e.g., *Eubacterium coprostanoligenes*, Oscillospiraceae, Ruminococcaceae), and a decline in fiber- and mucin-degrading bacteria like *Akkermansia* and Muribaculaceae. Functional predictions indicated upregulated amino acid metabolism and chitin degradation. Despite compositional shifts, microbial diversity remained stable, with no signs of dysbiosis. These findings suggest that *T. molitor* meal supports a safe, functional adaptation of gut microbiota to high-protein, chitin-rich diets, supporting its potential use in monogastric animal nutrition.

## 1. Introduction

Edible insects are gaining scientific interest as alternative protein sources due to their nutritional value, low environmental impact, and potential to address global food insecurity [1,2]. They provide diverse macro- and micronutrients [3] and may help meet protein demands in vulnerable populations [4]. Insect farming has high feed conversion efficiency, and lower greenhouse gas emissions, water use, and land requirements than traditional livestock [5,6,7]. Cultural acceptance and production scalability remain key challenges [8].

Edible insects present notable nutritional and functional advantages. According to Nowakowski et al. [9], edible insects are generally rich in essential amino acids, vitamins, and minerals. Mancini et al. [10] emphasize that insects can serve as viable alternatives or complements to conventional livestock proteins, contributing to a reduced environmental footprint of meat production. Zielińska et al. [11] report that several insect species, including *Tenebrio molitor*, provide particularly high-quality protein, suitable for human consumption and animal feed. Insects have protein content comparable to animal- and plant-based foods such as beef, eggs, milk, and soy, with all essential amino acids meeting WHO requirements [12,13]. Furthermore, Moruzzo et al. [14] highlight that incorporating insects into diets aligns with global sustainability efforts, demonstrating the potential of insect farming to support Sustainable Development Goals through efficient resource use. Importantly, the nutritional composition of edible insects is not uniform among species. Comparative estimates show wide ranges on a dry weight basis: approximately 35–60% (with higher values reported for some Orthoptera), 10–50% fat (larval stages tend to be higher), and 3–8% ash, along with species-specific profiles of essential minerals (e.g., Fe, Zn, Mg, Cu) and variable chitin content [9,10,11]. As emphasized by other authors, these differences reflect taxon, life stage, diet, and processing, and result in distinct amino acid and lipid profiles in commonly used edible insects [9,10,11]. Such heterogeneity has nutritional significance and may shape host responses, including gut microbiota structure and metabolism, when insects are used as feed or food ingredients. Accordingly, findings obtained with one insect species should not be generalized to others without considering species-level composition, as each may influence gastrointestinal homeostasis differently.

The gastrointestinal tract of mammals is populated by a microbiota with a different composition in individual animal species, which is related to their anatomical specificity and typical diet [15]. The intestinal microbiota is responsible for important biological functions such as: host nutrient metabolism, development and maintenance of the intestinal mucosal barrier, immunomodulation, protection against pathogens, xenobiotic and drug metabolism [16]. In addition, intestinal microorganisms break down dietary fiber and synthesize vitamins, serotonin, dopamine, and so called short-chain fatty acids (SCFAs) which help maintain homeostasis of the entire host organism.

Foods are mainly digested in the stomach and the small intestine, but indigestible food compounds and endogenous proteins secreted in the small intestine enter into the large intestine for microbial fermentation and putrefaction, which shape a diverse gut microbiota [17]. Therefore diet has been shown to be a key factor influencing the composition and function of the gut microbiota [18]. The composition of the microbiota may change depending on the content of macronutrients in the diet and even the caloric value of meals [19].

It should be noted that the insects are used whole, and along with the digestive tract, insect-specific microbiota may be introduced into the food. Klunder et al. [20] emphasize that the microbiological safety of insect-based products is crucial, as novel foods derived from insects may harbor microbial communities not commonly found in traditional livestock or plant-based products. Garofalo et al. [21,22] used high-throughput sequencing to characterize the microbiota of commercially available insect products, revealing a diverse assemblage of microorganisms. These studies highlight both a challenge and an opportunity inherent in insect-based diets, which must remain both safe and beneficial to health. Meijer et al. [23] discuss the evolving regulatory framework within the European Union, noting that more rigorous oversight is needed to ensure that insect-based products meet established safety standards. Ensuring safety of insect-based foods, including effective control of foodborne diseases, will be a significant milestone on the path to maintaining a sustainable food chain [24].

Insect derived components can also significantly influence gastrointestinal health. Stull et al. [25] demonstrate that even modest cricket (*Gryllodes sigillatus*) supplementation can modulate the human gut microbiota, potentially yielding beneficial shifts in microbial composition. Borrelli et al. [26] showed that insect-based diets can enhance SCFAs production and promote a healthier microbiome profile in laying hens. Similar observations have been reported in rodent studies, with Lanng et al. [2] finding that partial substitution of meat with insect protein in a carnivore diet influence both the gut microbiome and metabolome. These findings showed the growing body of evidence linking insect-based feeding strategies to measurable physiological outcomes.

Few studies have assessed the effects of insect protein on muscle protein synthesis and muscle mass, showing that insect protein is suboptimal compared to whey in both amino acid availability and stimulation of muscle protein synthesis [27,28]. In contrast, a study in pigs showed lower ileal digestibility of most amino acids in diets containing insect meal compared to a control diet and small changes in the metabolome when 10% of conventional protein was replaced with insect protein [29].

Despite these advances, major gaps remain regarding the long-term impact of feeding insects to various animal models, as well as the specifics of how high-inclusion insect diets affect gut microbiota composition and function. Studies focusing on companion or laboratory animals are particularly important for extrapolating potential health benefits or risks to other species [30].

Therefore, the aim of this study was to assess the impact of a diet based on *T. molitor* on the microbiota of laboratory rats. Standard microbiological methods were employed to validate *Lactobacillus* spp., Enterobacteriaceae, *Clostridium* spp., *Enterococcus* spp., *Bacteroides* spp., *Staphylococcus* spp., total aerobic bacteria, anaerobic bacteria, and fungi communities from individual fecal samples. Furthermore, to gain deeper insights into the microbial composition and potential functional shifts within the gut microbiome, metagenomic analysis using Next-Generation Sequencing was conducted at the end of the 9-week experimental period. This work aims to develop and safely implement *T. molitor* meal as a functional protein source in monogastric nutrition. In particular, presented research aims to support the formulation of insect-based diets for companion animals (e.g., dogs), and the generated gut-microbiome data may also be informative for future applications in human nutrition.

## 2. Results

### 2.1. Microbiological Analysis of Rats Feces

**Figure 1:**
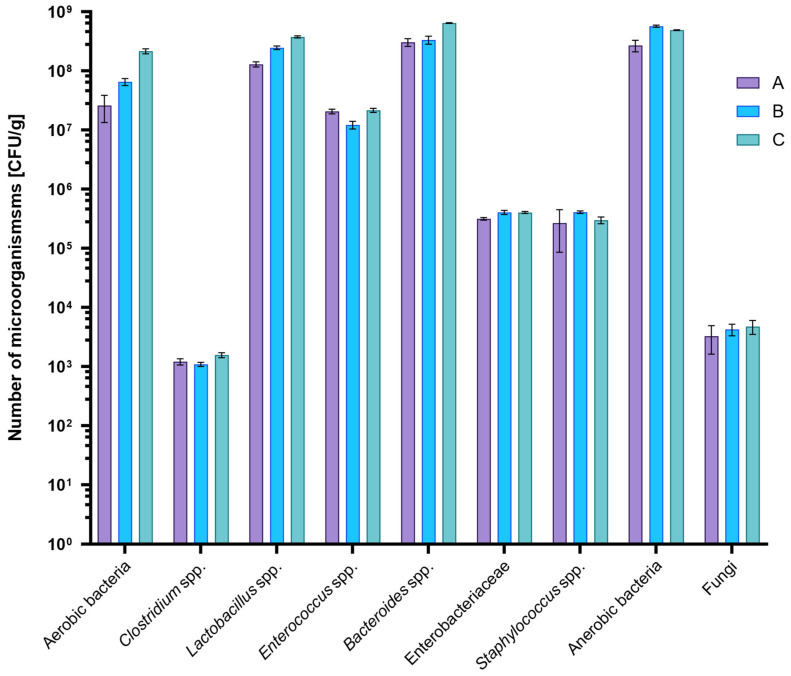
Average counts of selected microorganisms in rat feces by group: A (*n* = 15, 35% *T. molitor* meal), B (*n* = 15, 35% chicken meal), and C (*n* = 15, standard rat feed). Data are presented as mean values; error bars indicate the standard deviation (SD).

The average aerobic bacterial count was the lowest in Group A (2.59 × 10^7^ CFU/g), higher in Group B (6.52 × 10^7^ CFU/g), and the highest in Group C (2.15 × 10^8^ CFU/g) (Figure 1). Statistically significant differences were observed over time (weeks 4, 6, and 8) within any individual group (*p* < 0.05). Group A exhibited a significantly lower aerobic bacterial count over time compared to Groups B and C (Table S1). For *Clostridium* spp., the mean values ranged from 1.09 × 10^3^ CFU/g in Group B to 1.56 × 10^3^ CFU/g in Group C (Figure 1). In Group A, the *Clostridium* spp. population decreased significantly over the course of the experiment (*p* < 0.05). In contrast, Groups B and C showed an increase in *Clostridium* spp. counts after six weeks, followed by a subsequent decrease (Table S1). *Lactobacillus* sp. counts ranged from 1.29 × 10^8^ CFU/g to 3.74 × 10^8^ CFU/g within Group A; however, no statistically significant differences were detected among the three diet groups or across the 6- and 8-week time points (*p* > 0.05) (Figure 1, Table S1). *Enterococcus* spp. counts in all tested samples were broadly similar, ranging on average from 1.22 × 10^7^ to 2.15 × 10^7^ CFU/g depending on the rat group. Likewise, *Enterobacteriaceae* concentrations averaged between 3.15 × 10^5^ CFU/g (Group A) and 4.04 × 10^5^ CFU/g (Group B). Neither *Enterococcus* spp. nor *Enterobacteriaceae* levels differed significantly over time or among the three diet groups (*p* > 0.05) (Figure 1, Table S1). Similarly, *Bacteroides* counts exhibited no statistically significant variations across the different feeding regimens or sampling weeks, averaging between 3.04 × 10^8^ and 6.43 × 10^8^ CFU/g (*p* > 0.05) (Figure 1, Table S1). Coagulase-positive *Staphylococcus* counts ranged from a mean of 2.67 × 10^5^ CFU/g in Group A to 4.08 × 10^5^ CFU/g in Group B. A statistically significant increase in *Staphylococcus* genus was noted within Group A overtime (rising from 1.37 × 10^5^ CFU/g at week 4 to 4.74 × 10^5^ CFU/g at week 8). In Groups B and C, the counts remained stable yet were statistically higher than those observed in Group A (*p* < 0.05) (Figure 1, Table S1). In Groups B and C, the counts remained relatively stable but were significantly higher compared to Group A (*p* < 0.05) (Figure 1, Table S1). Regarding total anaerobic bacteria, the lowest mean count occurred in Group A (2.69 × 10^8^ CFU/g), followed by Group C (4.89 × 10^8^ CFU/g), and the highest count appeared in Group B (5.70 × 10^8^ CFU/g). In Group A anaerobic bacteria population was significantly lower than in the other two diet groups (*p* < 0.05) (Figure 1, Table S1). Finally, the overall fungal count ranged from 3.27 × 10^3^ CFU/g (Group A) to 4.74 × 10^3^ CFU/g (Group C). A statistically significant rise in fungal load over time was observed in Groups B and C, while Group A exhibited significantly lower fungal counts compared to these two groups (*p* < 0.05) (Figure 1, Table S1).

### 2.2. Microbial Community Compositional Analysis

Each sample yielded between ≈100,000 and 130,000 non-chimeric, high-quality reads. Classification efficiency remained high (over 70% of raw reads typically passed quality control), allowing robust comparisons among samples. Across all six datasets, a total of 179 bacterial genera from 15 distinct phyla were detected, with varying relative abundances based on the diets (A, B, or C). Additionally, Archaea (primarily Euryarchaeota) were consistently present at low levels (<1.0% in most samples).

Across all samples, the gut microbiota was dominated by the phyla Firmicutes, Bacteroidota, Verrucomicrobiota, and Actinobacteriota, with diet-specific shifts in their relative abundances. Firmicutes was the predominant phylum in every group, but rats on the high-protein diets (Groups A and B) showed a higher Firmicutes proportion (≈69–71% of sequences) than those on the standard diet (Group C, ≈48–51%). Correspondingly, Bacteroidota and Verrucomicrobiota were markedly elevated in Group C. Group C harbored ≈15–16% Bacteroidota and ≈19–30% Verrucomicrobiota, roughly 2–3 times the levels observed in the insect meal group (Group A: ≈9% Bacteroidota, ≈8–13% Verrucomicrobiota) and chicken meal group (Group B: ≈5% Bacteroidota, ≈8–18% Verrucomicrobiota). This disparity is reflected in the Firmicutes: Bacteroidota ratio, which was the highest in the chicken-fed rats (≈13:1) and the lowest in standard-fed rats (≈3:1). Actinobacteriota showed the opposite trend: Group B exhibited the greatest Actinobacteriota relative abundance (≈12% on average), about double that in Group A (≈6%) and higher than in Group C (≈5–10%). Minor phyla were also diet-dependent; for example, Desulfobacterota (class Desulfovibrionia) comprised a small fraction in all samples, but tended to be higher in the insect diet group (≈3–4%) compared to ≈2% in the other groups. Archaea (family Methanobacteriaceae, phylum Euryarchaeota) were detected at low levels (<1%) in all groups, with a slight increase in the chicken-based diet (Group B ≈ 0.97% vs. Group A ≈ 0.5% and Group C ≈ 0.2%). At finer taxonomic levels, each diet fostered a distinct community profile. In Group C (standard diet), the expansion of Verrucomicrobiota was driven by an especially high abundance of the mucin-degrading genus *Akkermansia*. *Akkermansia* constituted ≈20–25% of the total community in Group C. This represents a ≈2–3-fold increase in *Akkermansia* spp. relative to the *T. molitor* and chicken meal groups, where it accounted for ≈8–13% of sequences. Likewise, the higher Bacteroidota in Group C corresponded to a greater representation of the family Muribaculaceae (order Bacteroidales), a dominant bacterial family in rodent guts. Muribaculaceae (often represented as the Muribaculaceae genus due to unclassified members) averaged ≈11% in standard-fed rats, double the relative abundance in Group A (≈3–4%) and about fivefold higher than in Group B (≈2%). Group C also showed an enrichment of *Bifidobacterium* spp. (family Bifidobacteriaceae) compared to the protein-based diets. In Group C, *Bifidobacterium* spp. reached ≈7.3% of the community, whereas in Group A it remained <1% and in Group B it was nearly absent (e.g., *Bifidobacterium animalis* < 0.5% in B-1). These taxa suggest that the fiber-rich standard diet promoted well-known fermentative genera (Muribaculaceae, *Bifidobacterium*) and mucin utilizers (*Akkermansia*).

In contrast, the high-protein diets (Groups A and B) favored different Firmicutes clades and Actinobacteria. Group B (35% chicken meal) was characterized by a greater prevalence of Actinobacteria from class Coriobacteriia. This included the families Coriobacteriaceae and Eggerthellaceae (genera such as *Collinsella* and *Adlercreutzia*), which together contributed ≈8–12% of the community in Group B (versus ≈3–6% in Group C). Although bifidobacteria were low in Group B, other Actinobacteria associated with protein fermentation were relatively enriched. The chicken diet also resulted in a notable expansion of Erysipelotrichaceae, a family of Firmicutes linked to high fat/protein intake. In particular, the genus *Allobaculum* (*Erysipelotrichaceae* family) was prominent in Group B, reaching ≈7.7% in one sample (and ≈3.5% in the other). On average *Allobaculum* spp. Was higher in Group B than in either Group A (where it was detected at lower abundance) or Group C. Another Erysipelotrichaceae genus, *Dubosiella*, was also abundant in Group B (≈5–8%) and present in all diets (ranging 4–7% in individuals across groups). The Peptostreptococcaceae family (order Peptostreptococcales-Tissierellales) was a major Firmicutes component in all groups, but *Romboutsia* spp. (a genus in this family) showed diet-related variation. *Romboutsia* spp. Was prevalent in Group C (averaging ≈13–14%), and somewhat lower in Group B (≈8–10%) and Group A (≈4–5%). Inversely, Group A showed the highest levels of certain Oscillospirales and Ruminococcaceae clades. Notably, the *Eubacterium coprostanoligenes* group (an Oscillospirales lineage involved in cholesterol metabolism) was enriched by the *T. molitor* diet, representing ≈9% of the community in Group A compared to ≈4% in Group B and ≈3.6% in Group C. Group A also tended to harbor more Oscillospiraceae UCG-005 (another genus in Oscillospirales; ≈6% in A vs. ≈2–4% in B and C), and more *Ruminococcus* spp. (family Ruminococcaceae) than the other diets. Meanwhile, Christensenellaceae (order Christensenellales) were consistently present (≈5–6% across all groups) and did not vary greatly with diet.

**Figure 2:**
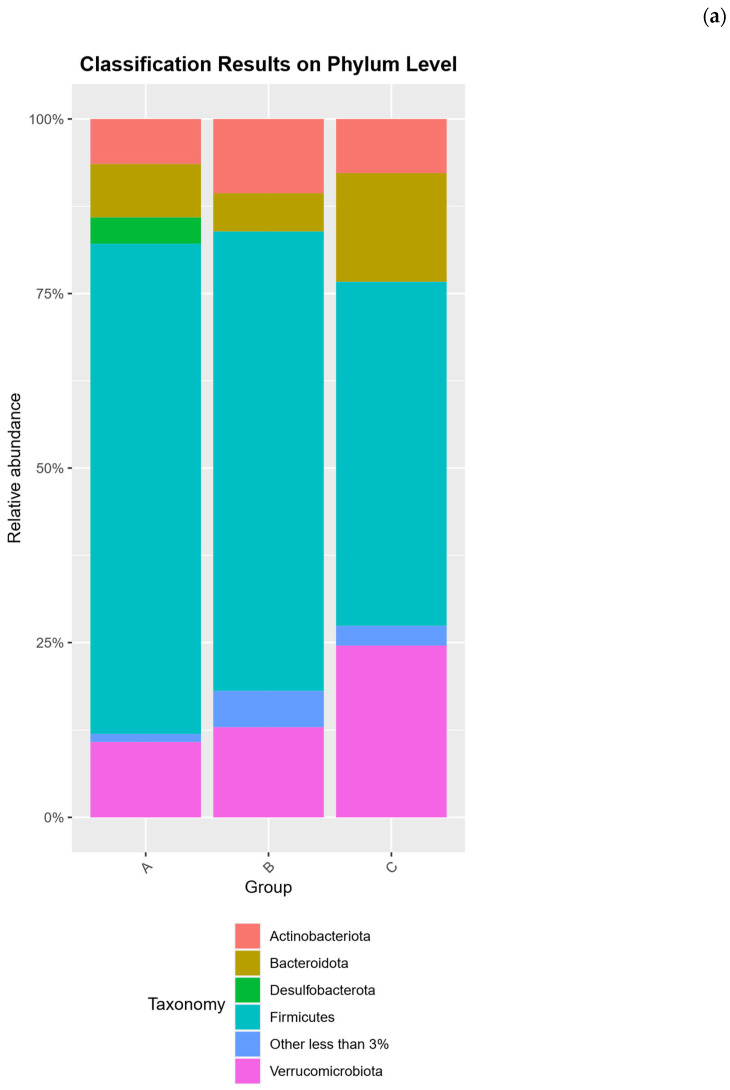

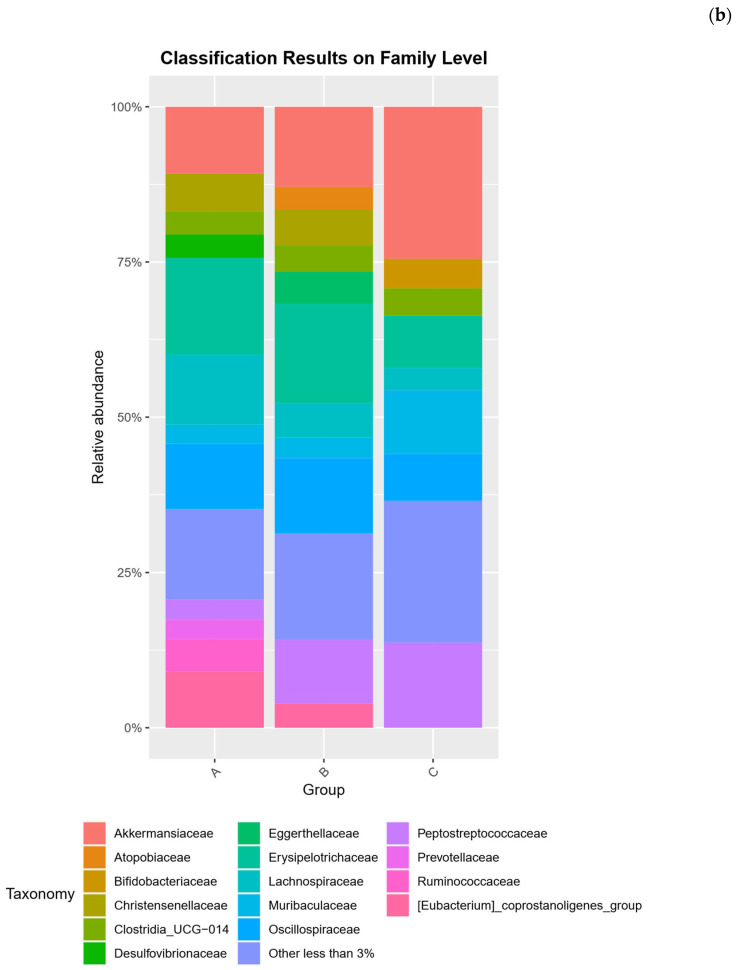

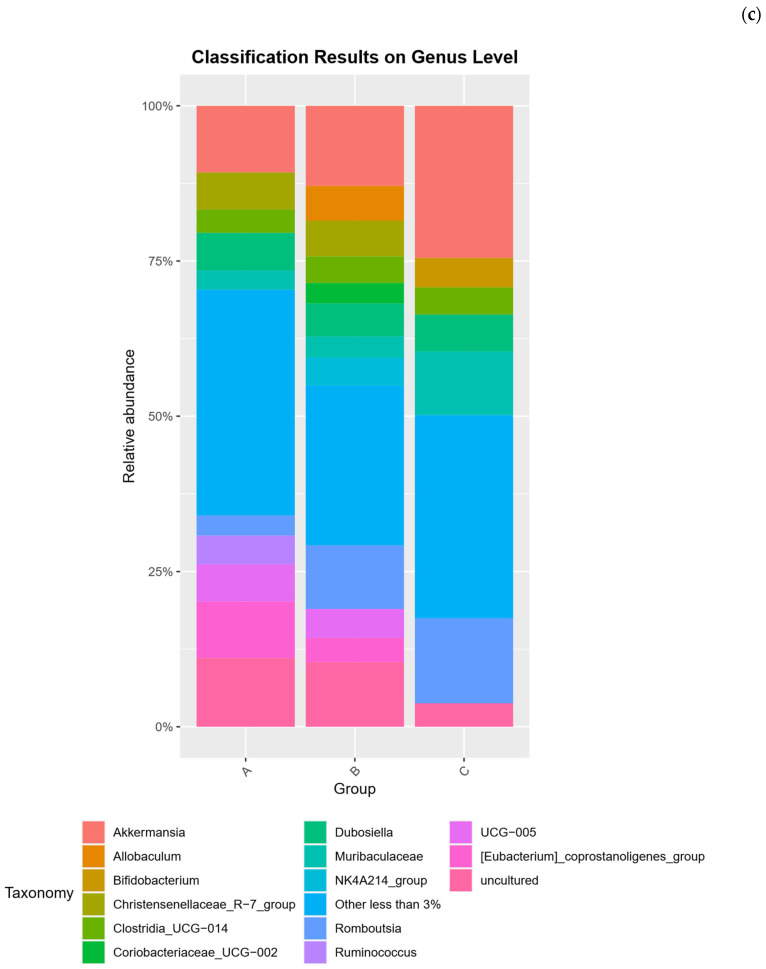
The composition analysis of the microbiota community at the (**a**) phylum, (**b**) family and (**c**) genus level. A (*n* = 15, 35% *T. molitor* meal), B (*n* = 15, 35% chicken meal), and C (*n* = 15, standard rat feed).

Overall, these data indicate that the standard rat feed (Group C) fostered a gut community with greater proportions of carbohydrate- and mucus-degrading bacteria (Muribaculaceae, *Bifidobacterium* spp., *Akkermansia* spp.), whereas both protein-rich diets shifted the community toward Firmicutes-dominated profiles with more protein/fat-associated taxa. The insect meal diet in particular enriched specific Clostridial groups (Oscillospirales such as the *Eubacterium coprostanoligenes* clade), while the chicken meal diet preferentially expanded Actinobacteria (Coriobacteriia) and *Erysipelotrichaceae* (e.g., *Allobaculum* spp.). Despite these trends, many core taxa (e.g., clostridia from the families *Lachnospiraceae*, *Oscillospiraceae*, and *Ruminococcaceae*) were common to all diets, and no taxa was entirely exclusive to one diet. Differences were therefore largely in relative abundance rather than presence/absence. Importantly, at the OTU level (97% similarity phylotypes), similar patterns emerged: the most abundant OTUs in Group C were classified as *Akkermansia* spp. (accounting for ≈24% of reads on average in C) and uncultured *Muribaculaceae*, whereas Group A’s top OTUs included members of the *Eubacterium coprostanoligenes* group and *Dubosiella* spp., and Group B was enriched in OTUs affiliating with *Allobaculum* spp. and Coriobacteriaceae. These OTU-level distinctions mirror the genus-level trends above, proving that diet substantially alters the gut community structure across all taxonomic ranks. The composition analysis of the microbiota community at the phylum, family and genus level is provided in Figure 2.

### 2.3. Alpha Diversity Analysis

**Figure 3:**
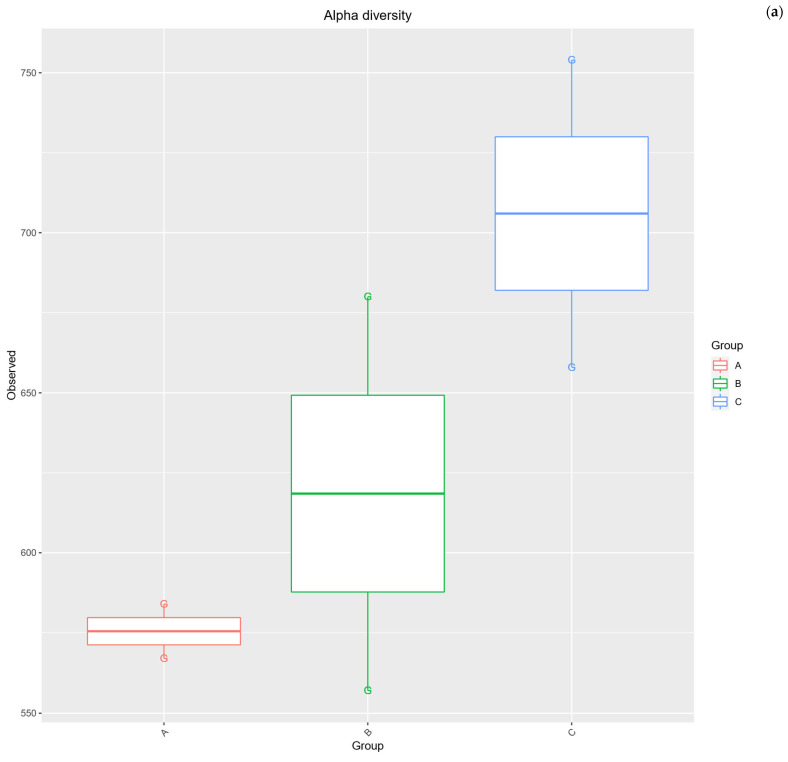

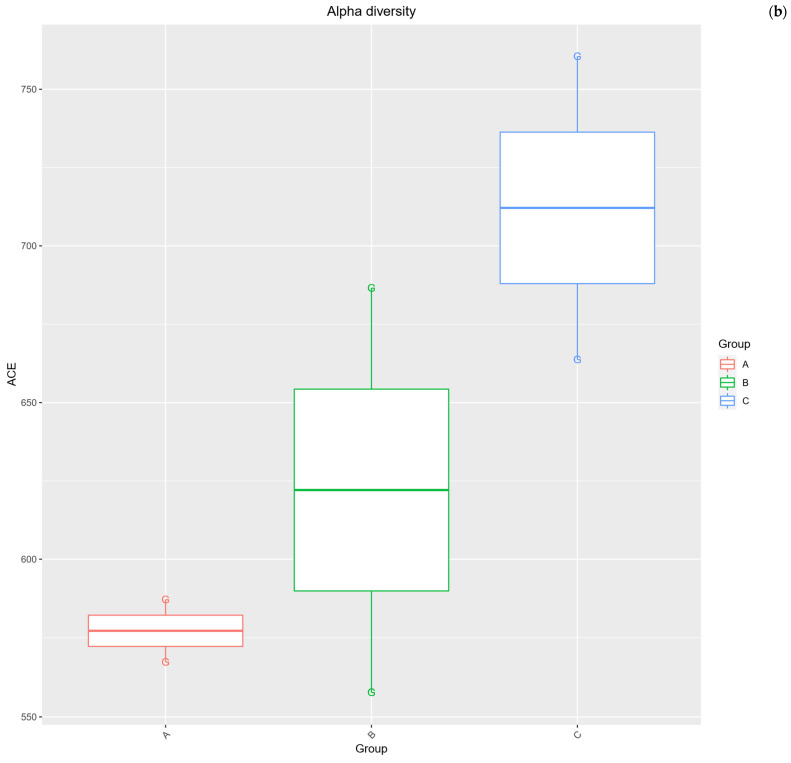

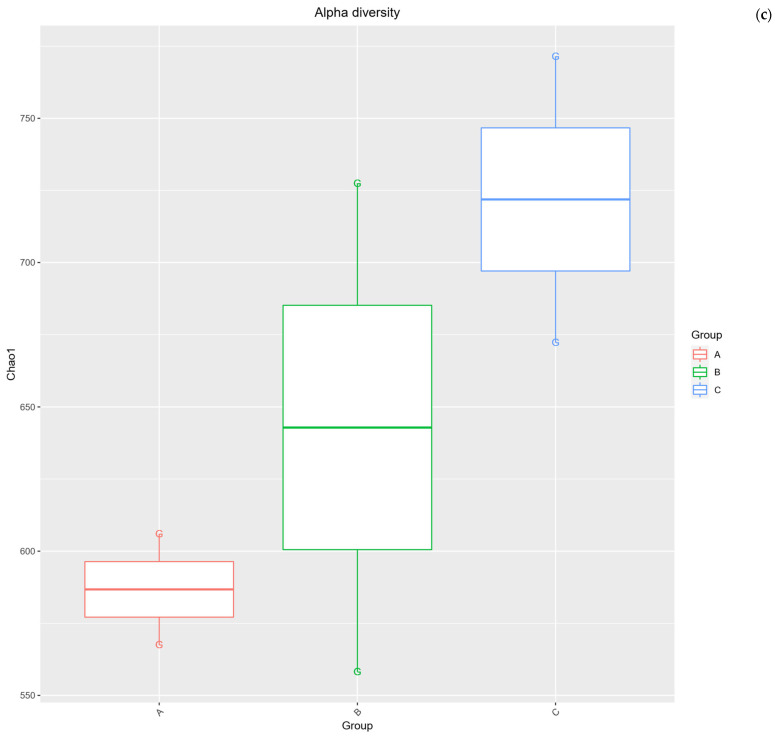

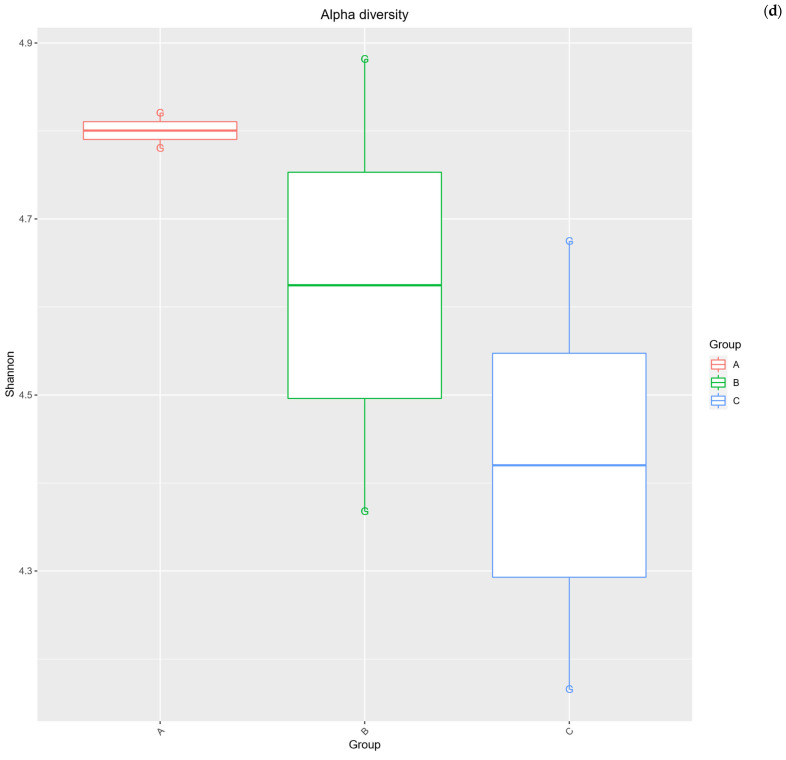

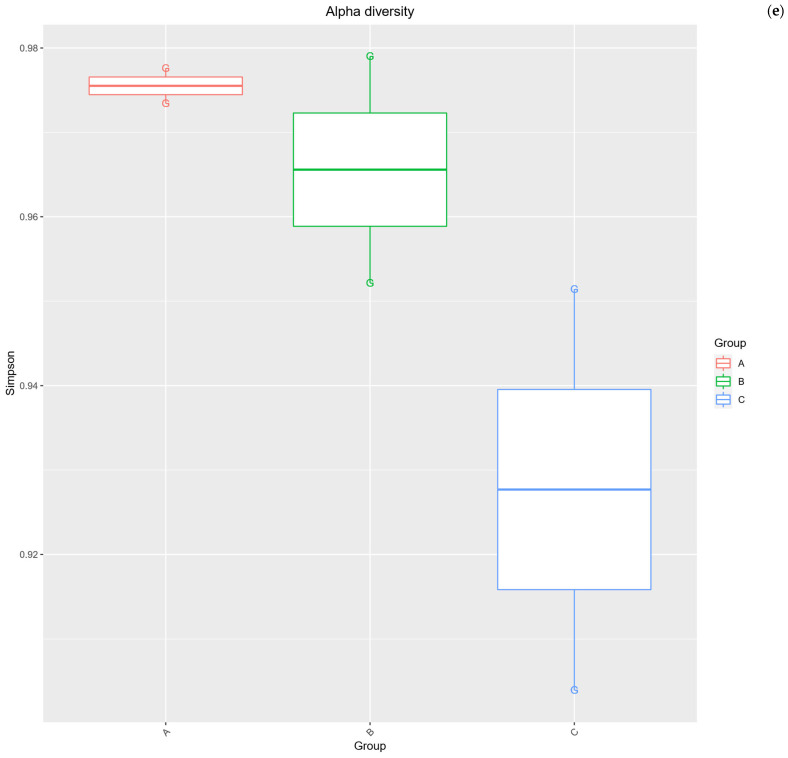

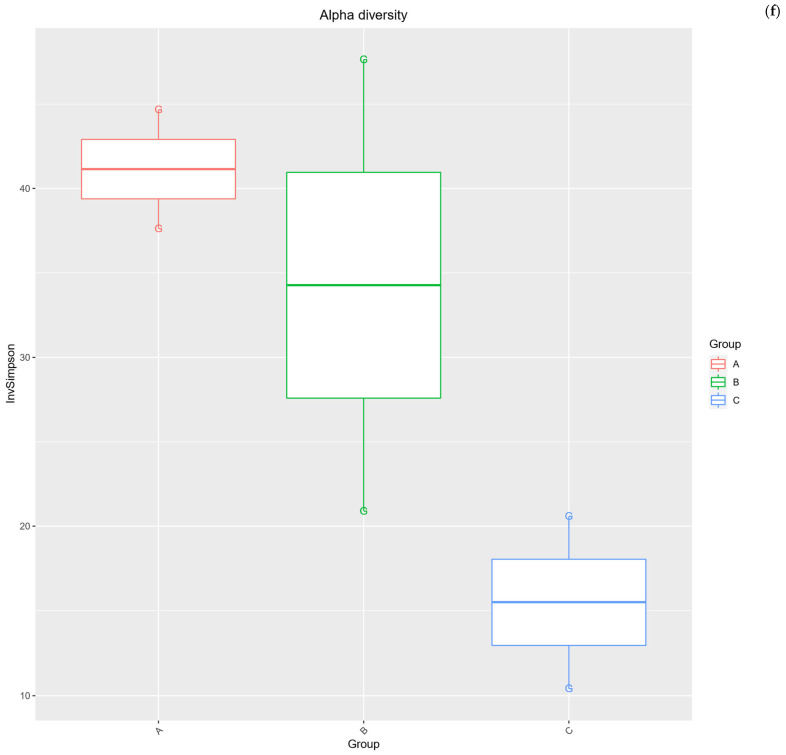
Box-plots for alpha diversity including (**a**) Observed OTU, (**b**) ACE, (**c**) Chao1, (**d**) Shannon, (**e**) Simpson and (**f**) Inverted Simpson within studied groups: A (n = 15, 35% *T. molitor* meal), B (n = 15, 35% chicken meal), and C (n = 15, standard rat feed). The boxes illustrate the interquartile range, between the first (Q1) and third quartiles (Q3), with the line inside marking the median. The bars extend to the smallest and largest values that fall within 1.5 times the interquartile range from the lower and upper quartiles.

Measures of species richness—including Observed OTUs and richness estimators (Chao1 and ACE)—were slightly higher in the standard diet group but showed considerable overlap with the high-protein groups. For example, the mean number of observed OTUs in Group C was ≈706, compared to ≈576 in Group A and ≈619 in Group B, but this difference was not statistically significant (pairwise *t*-tests, *p* > 0.2 for both comparisons). Similarly, the Chao1 estimator trended higher in Group C (mean ≈ 722) than Group A (≈587) or B (≈643), suggesting Group C may harbor more total phylotypes, but again the variation was large (*p* > 0.19 for A vs. C) and prevented any significant detection. Measures of community diversity that account for richness and evenness (Shannon’s diversity H′ and Simpson’s index) showed no significant diet effect either. Shannon indices were in a narrow range across all diets (approximately 4.4–4.8), indicating comparable overall diversity in the three groups (for instance, Shannon mean 4.80 in Group A vs. 4.62 in Group B; *p* = 0.62). Simpson’s index (1–D) remained high in all samples (mean ≈0.93–0.98), reflecting that dominant taxa did not completely dominate the community. There was a slight trend toward lower evenness in the standard-fed rats: Group C had the lowest Simpson’s diversity (mean 0.928 vs. 0.966 in Group B and 0.976 in Group A), corresponding to one taxon (*Akkermansia* spp.) dominating a larger share of the community. This was evident in the inverse Simpson index as well—a metric of diversity where higher values indicate greater evenness—which was almost three-fold lower in Group C (15.5) compared to Group A (41.1). Although this difference approached significance (*p* = 0.065 for inverse Simpson, A vs. C), it did not meet the 0.05 threshold. In sum, all alpha diversity metrics consistently suggested that no diet produced a statistically significant gain or loss of diversity relative to the others. The insect meal and chicken meal diets-maintained diversity on par with the standard diet, with only minor non-significant shifts (the Group C tended to have more OTUs but slightly lower evenness due to *Akkermansia* spp.). These results indicate that, despite impact on community composition, the alternative protein diets did not cause a collapse or major expansion in overall within-host diversity of the gut microbiome. Detailed data are provided in Figure 3.

### 2.4. Beta Diversity Analysis

Differences in community composition between the diet groups were evident, although not statistically significant given the sample size. Overall, microbial communities tended to cluster by diet, indicating that rats with the same diet harbored more similar microbiota to each other than to rats on different diets. Pairwise PERMANOVA comparisons showed large effect sizes of diet on community composition: for example, comparing Group A vs. Group C yielded an R^2^ ≈ 0.86, and Group B vs. Group C had an R^2^ of ≈0.72. Group A vs. B (the two high-protein diets) showed a similarly high R^2^ ≈ 0.74. These values suggest substantial community shifts in response to the different diets. However, because of limited replication, none of these differences reached significance under PERMANOVA (*p* = 0.333 for all pairwise tests). In practical terms, the high variability and low degrees of freedom meant that diet-related clustering did not achieve statistical reliability, even though the magnitude of compositional differences was biologically large.

**Figure 4:**
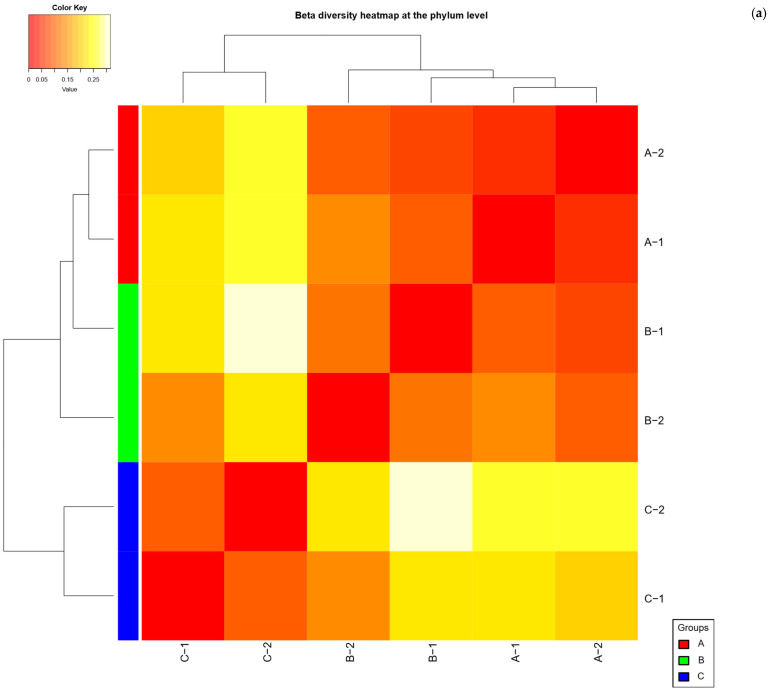

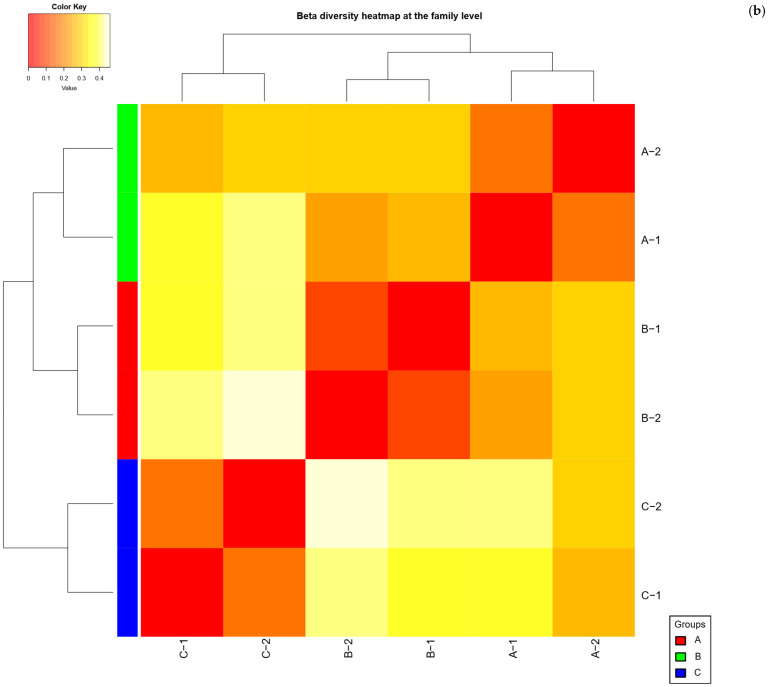

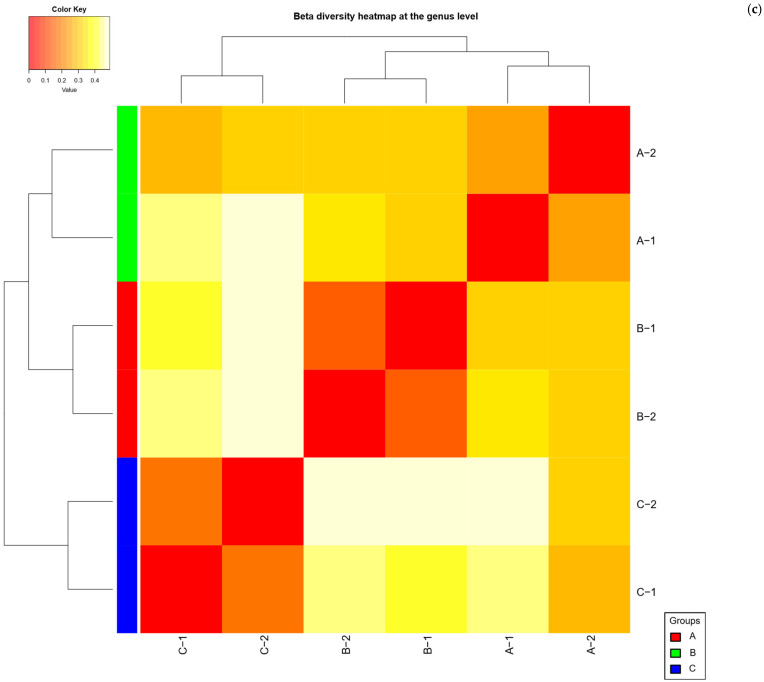
Beta diversity heatmap of (**a**) phylum, (**b**) family and (**c**) genus for studied groups. A (n = 15, 35% *T. molitor* meal), B (n = 15, 35% chicken meal), and C (n = 15, standard rat feed).

Examining the beta diversity patterns more qualitatively, we observed that the Group C microbiomes were the most distinct, while the two high-protein diet groups (A and B) were somewhat more similar to each other. Bray–Curtis distances between Group C samples and either high-protein group were larger than distances between Group A and B. This aligns with the taxonomic data showing that Groups A and B shared many features (both dominated by Firmicutes with lower Bacteroidota), whereas Group C differed by having much higher Bacteroidota and *Akkermansia* spp. Indeed, the greatest community divergence was between Group A and Group C (PERMANOVA F = 12.075, R^2^ = 0.858, *p* = 0.333), followed by Group B vs. C (F = 5.18, R^2^ = 0.721, *p* = 0.333). Group A vs. B had the smallest divergence (F = 5.652, R^2^ = 0.739, *p* = 0.333), suggesting the insect and chicken diets resulted in more alike microbiota compositions relative to the difference between either one or the standard diet. Any significant clustering by diet were not detected, when using other distance metrics (e.g., unweighted UniFrac gave similar outcomes), confirming that diet-driven differences, while apparent, could not be confirmed statistically due to the small sample counts. Nonetheless, the beta diversity trends consistently pointed to diet as an important driver of community composition: microbial communities of rats feeding on *T. molitor* vs. chicken vs. standard rat feed formed three separable clusters in multivariate space, with overlapping clusters for the two alternative protein diets and a clearly separated cluster for the standard diet group. Data are visualized in Figure 4.

### 2.5. PCA and PCoA Ordination

The ordination plots revealed a clear segregation of samples according to diet groups, in agreement with the PERMANOVA results. In the PCoA plot based on Bray–Curtis dissimilarities, the first principal coordinate (PCo1) accounted for the majority of between-sample variation and primarily separated the standard diet samples from the high-protein diet samples. Group C samples clustered together on one end of PCo1, distinctly apart from the cluster formed by Groups A and B. This indicates that the overall community profiles of standard-fed rats were different from those of rats on either *T. molitor* or chicken diets. The separation along PCo1 was driven by the taxa that differed most by diet: samples with high *Akkermansia* spp. and Muribaculaceae (i.e., Group C) scored at one extreme of PC1, whereas samples enriched in Firmicutes (clostridia/Erysipelotrichia) and Coriobacteriia (Groups A and B) occupied the opposite end. The second principal coordinate (PCo2) explained a smaller portion of the variance and tended to distinguish Group A vs. Group B within the high-protein cluster. Along PCo2, the *T. molitor* meal samples separated modestly from the chicken meal samples. This suggests that although both alternative diets shifted the microbiota in a similar general direction (as seen on PC1), there were secondary differences between *T. molitor*-based and chicken-based protein sources. Specifically, Group B (chicken) samples were associated with higher scores on PCo2 in correspondence with greater Actinobacteria (Coriobacteriaceae) and *Allobaculum* spp. abundance, whereas Group A samples had lower PCo2 scores linked to higher *Eubacterium coprostanoligenes* group and Oscillospiraceae UCG-005. These subtle differences along PC2 align with the genus-level variations noted between Groups A and B. Overall, the PCoA ordination illustrates that dietary regimen imposes a structured variation on the gut microbiome: replicates from the same diet cluster together, and the ordination axes reflect the gradients of key microbial taxa shaped by each diet. The PCA on taxonomic composition (e.g., at the genus level) further confirmed these patterns. In a PCA biplot of the top genera, PC1 explained a large portion of the variance (on the order of ≈50%) and clearly separated the standard diet group from the protein-supplemented groups. PC1 loadings indicated that *Akkermansia* genus, Muribaculaceae (family), and *Bifidobacterium* genus were strongly positively associated with PC1 (driving the Group C positions), whereas genera characteristic of high-protein diets (such as *Allobaculum* spp., *Dubosiella* spp., and the *Eubacterium coprostanoligenes* group) loaded negatively on PC1 (driving Groups A/B). PC2 of the PCA (explaining ≈20% of variance) captured the divergence between the two high-protein diets: *Allobaculum* spp. and several genera within Coriobacteriia (enriched in Group B) had a strong influence on one end of PC2, whereas Group A was associated with greater contributions from Oscillospiral taxa and Desulfovibrionaceae. This separation was modest, reflecting that Groups A and B shared many taxa in common, yet it was sufficient to distinguish the *T. molitor* vs. chicken diet samples in ordination space. The clustering patterns in both PCA and PCoA were consistent—points representing Group C were isolated from the others, while Groups A and B formed adjacent but distinct clusters. Notably, the distance between Group C and the others on the primary axis was larger than the distance between Group A and B, illustrating again that the largest shift in microbial community structure was caused by the absence vs. presence of dietary fiber (standard rat feed vs. high-protein diets), with a smaller but noticeable effect of protein source (insect vs. chicken) on the microbiome. These multivariate analyses, therefore, provide a confirmation that diet type structured the gut microbial communities, even though inferential statistics (due to low sample number) did not declare significance. The ordination results are in line with expectations from the taxonomy findings: diets cluster together because they promote specific bacterial populations—e.g., standard diet clustering driven by fiber-fermenters and mucolytic bacteria, versus protein diets clustering driven by amino acid- and fat-associated bacteria.

### 2.6. Heat Tree Visualization

Heat tree visualization was employed to compare the taxa abundances across diets in a hierarchical manner. In the heat tree comparing Group C vs. Group A, the branches corresponding to phylum Bacteroidota and Verrucomicrobiota were higher in abundance on the Group C side, whereas many Firmicutes branches glowed on the Group A side. For instance, the Bacteroidales clade (within Bacteroidota) was enlarged in Group C, reflecting the higher Muribaculaceae in standard diet rats, while the Verrucomicrobiales branch (includes *Akkermansia* genus) was also clearly expanded in Group C. In contrast, on the *T. molitor* diet side of the heat tree, several clostridial orders were highlighted. The Oscillospirales branch (covering Ruminococcaceae and Oscillospiraceae, including the *Eubacterium coprostanoligenes* group) was more prominent in Group A, consistent with its enrichment in those samples. Likewise, the Erysipelotrichales branch (family Erysipelotrichaceae, genus *Dubosiella* and others) showed greater relative abundance in Group A in the heat tree comparison with Group C. These visual cues align with our quantitative findings that Group A had a higher representation of certain Firmicutes at the expense of Bacteroidota and Verrucomicrobiota compared to Group C.

The heat tree for Group B vs. Group C showed a similar overall pattern, with Group C again enriched in Akkermansiaceae and Muribaculaceae lineages. On the Group B side, the tree highlighted Actinobacterial clades—notably the Coriobacteriales branch (which includes Coriobacteriaceae/Eggerthellaceae). This indicates that Group B had a greater abundance of Coriobacteriia (as we observed with *Collinsella* and related genera) relative to Group C. Additionally, the Erysipelotrichaceae node was emphasized in Group B compared to Group C, corresponding to the expansion of *Allobaculum* spp. in the chicken diet. A direct comparison of the two high-protein diets (Group A vs. Group B heat tree) revealed more subtle differences. The heat tree identified a higher abundance of Coriobacteriales (Actinobacteria) and Erysipelotrichales in Group B, versus a higher abundance of specific Oscillospirales taxa in Group A. For example, nodes representing the family Eggerthellaceae (which includes protein-fermenting Actinobacteria) were larger in Group B, while the Oscillospiraceae/Ruminococcaceae nodes (including the cholesterol-degrading *Eubacterium* group) were larger in Group A. Though these differences were not statistically significant after correcting for multiple comparisons (given the small sample size), the heat tree clearly delineated the taxonomic signatures unique to each diet: Group A’s signature included more of certain clostridia (Oscillospiral OTUs), Group B’s signature included more Actinobacteria and *Allobaculum*, and Group C’s signature was dominated by *Akkermansia* genus and Muribaculaceae family. Heat tree visualizations for each group are presented in Figure S1.

### 2.7. Predicted Functional Shifts in the Gut Microbiome of Rats

Rats fed the insect-based diet (Group A) exhibited gut microbiome functions characteristic of both elevated-protein fermentation and complex carbohydrate degradation. This diet, rich in protein and chitin, elicited a microbial response marked by increased proteolytic activity alongside glycan turnover. The high abundance of protein-fermenting taxa (e.g., certain Firmicutes) indicates active amino acid catabolism, as undigested dietary protein promotes the proliferation of bacteria capable of degrading amino acids into nitrogenous end-products. Accordingly, metabolic pathways involved in branched-chain and aromatic amino acid degradation were likely upregulated, consistent with prior studies demonstrating modulation of amino acid metabolism by insect-based diets in the gut microbiome [31]. Concurrently, the presence of chitin stimulated microbial carbohydrate metabolism directed towards amino sugar utilization. Taxa capable of chitinolysis (e.g., some Bacteroidetes or specialized Firmicutes) would thrive by breaking chitin down into N-acetylglucosamine subunits. Members of the Bacteroidetes are known to harbor diverse polysaccharide utilization loci enabling them to degrade unusual glycans like chitin [32]. Thus, Group A’s microbiome likely carried an enriched capacity for amino sugar and nucleotide sugar metabolism, reflecting the conversion of *T. molitor*-derived chitin into fermentable sugars. This is consistent with findings that adding chitin-rich insect material shifts the gut community toward bacteria associated with fiber degradation and gut health.

**Figure 5:**
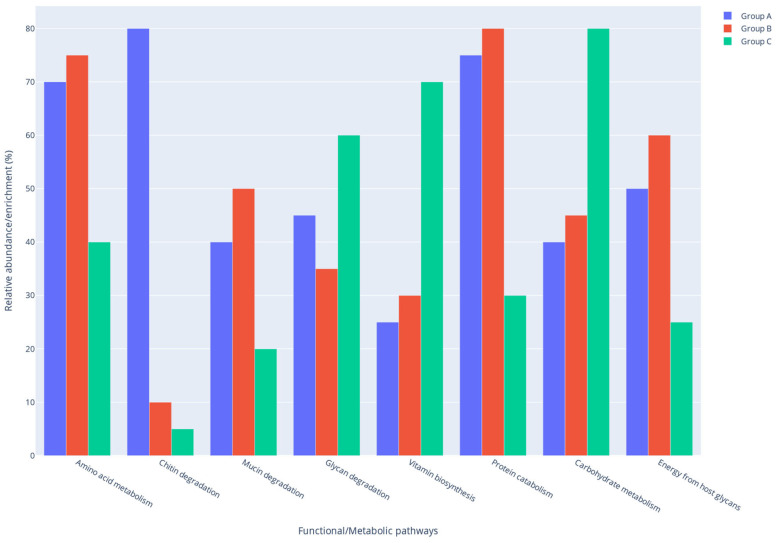
Predicted Functional Shifts in the Gut Microbiome of Rats fed with the insect-based diet A (n = 15, 35% *T. molitor* meal), B (n = 15, 35% chicken meal), and C (n = 15, standard rat feed).

The Group B, high in animal protein but low in fermentable fiber, favored microbial functions centered on protein utilization and alternative carbon sources from host glycans. The microbiota in this group was directed towards enhanced proteolysis and amino acid fermentation, evidenced by an abundance of proteolytic clostridia (e.g., Peptostreptococcaceae, Erysipelotrichaceae) and the relative shortage of carbohydrate specialists. Undigested proteins in the gut promote the growth of proteolytic bacteria, which convert amino acids into metabolites such as branched-chain fatty acids (BCFAs) precursors, ammonia, and hydrogen sulfide. Accordingly, pathways for amino acid degradation (e.g., lysine, tyrosine and tryptophan catabolic routes) and enzymes for peptide breakdown were elevated in Group B. This diet probably upregulated KEGG pathways related to energy metabolism from amino acids, aligning with the idea that a protein-centric microbiota will use amino acids as carbon and energy sources in the absence of dietary carbohydrates. Because the chicken diet lacked substantial fiber, the microbiota also scavenged host-derived glycans for energy. Mucin turnover was a key strategy: microbes in Group B would degrade the mucin glycoproteins secreted in the gut lining as an alternative nutrient source. Consistent with this, mucin-degrading taxa (e.g., *Akkermansia*, a genus within the phylum Verrucomicrobiota) were detected and likely more metabolically active in this low-fiber context. Functional inference suggests an increased expression of glycosidases and sulfatases targeting mucin O-linked sugars, falling under microbial glycan degradation pathways. Overall, Group B showed a functional profile dominated by protein and peptide turnover and host-glycan foraging. In contrast to the insect diet, little emphasis was on complex plant polysaccharide breakdown or vitamin biosynthesis in this group. Instead, the chicken-meal microbiome prioritized pathways for proteolytic metabolism and possibly stress responses to protein fermentation by-products (e.g., processing of sulfur-containing amino acids to H_2_S). This highlights a shift toward a more putrefactive metabolic profile when fermentable fiber is scarce. The summary of predicted functional shifts in the gut microbiome of rats for the three study groups is provided in Figure 5.

## 3. Discussion

The balance of the intestinal microbiota is an important factor determining the homeostasis of the host organism. The gastrointestinal tract of healthy humans and animals is predominantly colonized by autochthonous microorganisms that are either neutral or beneficial to host health [33]. However, excessive proliferation of bacteria classified as neutral may disrupt the systemic function. *Enterococcus* spp., *Streptococcus* spp., *Bacteroides* spp., and *Escherichia coli* strains, which are naturally present within the large intestine, may cause diseases when becoming predominant [34,35]. In the current study, for most of the microorganism groups studied (*Lactobacillus* spp., Enterobacteriaceae, *Clostridium* spp., *Enterococcus* spp., *Bacteroides* spp.), no statistically significant differences were found in the concentration in the feces of rats regardless of their diet. However, statistically fewer aerobic bacteria, anaerobic bacteria, coagulase positive *Staphylococcus* spp. and fungi were noted in the group fed *T. molitor*-based feed compared to the other two studied feeding variants. Diet is one of the major factors affecting the quality of intestinal microorganisms [18,36]. However, the quantity and quality of intestinal microbiota is also determined by environmental conditions, overall health, stress and individual factors (age, gender, genotype, intestinal passage time and peristalsis) [36].

Comparison of the results obtained with existing literature is challenging due to the limited number of studies employing a similar experimental setup. No established reference values exist for individual groups of microorganisms, owing to numerous factors influencing the intestinal microbiome, including individual variability. Brooks et al. [37] found from 7.9 × 10^6^ CFU/g to 4 × 10^11^ CFU/g of anaerobic bacteria in rat feces depending on the culture medium (L10, BHI, MRS, Beerens medium). In the current study, the number of *Enterococcus* spp. in rat feces was found to be on average 1.22 × 10^7^–2.15 × 10^7^ depending on the feeding method. Chettaoui et al. [38] found high concentrations of enterococci in feces in rats fed a standard diet, in the order of 10^8^ CFU per gram. In humans, *Enterococcus* spp. generally constitute less than 1% of the intestinal microbiota and are present in feces, generally from 10^4^ to 10^6^ bacteria per gram [39]. In previous studies, the concentration of *Lactobacillus* spp. in rat feces was recorded at the level of 10^8^ (CFU)/g [38], as observed in the present study. In human, chicken and pig feces, *Lactobacillus* spp. are usually present in concentrations of 5.5 × 10^10^ (CFU)/g feces, 4.7 × 10^8^ (CFU)/g feces and 9.7 × 10^8^ (CFU)/g feces, respectively [40]. It should be emphasized that *Lactobacillus* spp. are generally considered non-pathogenic intestinal microorganisms with probiotic potential. Brooks et al. [37] showed that with the exception of the lactobacilli, the cultured isolates demonstrated low species diversity and poorly reflected the population, as defined through comparative sequence analysis. This justifies the use of the next-generation sequencing methods to study microbiota biodiversity. Fungi constitute approximately 0.1% of the gastrointestinal microorganisms, and under normal conditions complex interactions—including antagonism, synergy, or symbiosis occur between fungi, bacteria, and viruses within the animal gut. The total number of fungi increases from the ileum to the colon and reaches the highest density in the distal intestine of most monogastric animals [41,42]. Although extensive studies have been conducted on the bacterial community in the human and animal gut, research efforts to investigate the fungal diversity in monogastric animals are very limited. Only a limited number of intestinal fungal species can be identified and classified using traditional culture-dependent methods, due to the fastidious growth requirements of many fungi [42,43].

Analysis at the genus level showed that in group A the proportion of the following genera *Akkermansia*, *Romboutsia* and *Lactobacillus* in feces decreased compared to the B and C groups. On the other hand, the proportion of *Eubacterium coprostanoligenes* group, *Dubosiella* spp., UCG-005, Christensenellaceae R-7 group, *Ruminococcus* spp., clostridia UCG-014, *Faecalibaculum* spp., *Marvinbryantia* spp., *Collinsella* spp., *Desulfovibrio* spp., Lachnospiraceae NK4A136 group and NK4B4 group, *Prevotella* spp., *Bifidobacterium* spp., uncultured genera from Desulfovibrionaceae, Lachnospiraceae and Oscillospiraceae increased. In the case of NK4A214 group (family Oscillospiraceae), *Allobaculum* spp., *Adlercreutzia* spp., a smaller share was observed in group A than in B, but larger than in group C. *Bacteroides* spp. and *Methanobrevibacter* spp. occurred in the same percentage in groups A and C, at the same time larger than in group B. The remaining genera accounted for <1%.

The share of *Akkermansia* spp. in rats fed insect protein is interesting. The species *Akkermansia muciniphila* was identified, which occurred in the same percentage in groups A and B (0.4%) and almost twice as high in group C (0.8%), and another unidentified species, which in the studied groups A, B, C accounted for 10.4%, 12.5% and 23.7%, respectively. *Akkermansia muciniphila* is one of the most important species reported to be involved in the complex links between diet, metabolic and inflammatory diseases [44]. The relative abundance of *A. muciniphila* is inversely correlated with obesity in humans, and it was shown to alleviate insulin resistance and obesity while increasing gut barrier function in a mouse model of diet-induced obesity [19,44]. Its potential as a next-generation probiotic in the battle against metabolic disorders was confirmed in a first intervention trial targeting humans with metabolic syndrome and obesity [45]. In humans, *Akkermansia* candidate species display ecological co-exclusion, diversified functional capabilities, and distinct patterns of associations with host body mass. Long-term high-calorie diets have been shown to promote a reduction in the relative abundance of *Akkermansia* spp. and an increase in the pathobiont *Bilophila* spp. [46].

*Romboutsia ilealis* was found to be a natural and abundant inhabitant of the rat small intestine, specifically of the ileum [47]. Literature showed that the genus *Romboutsia* spp. covers a broad range of metabolic capabilities with respect to carbohydrate utilization, fermentation of single amino acids, anaerobic respiration and metabolic end products. Main differences between strains were found in their abilities to utilize specific carbohydrates, to synthesize vitamins and other cofactors, and their nitrogen assimilation capabilities. In addition, differences were found with respect to bile metabolism and motility-related gene clusters [48].

Although the gut communities in all groups were dominated by typical phyla (Firmicutes and Bacteroidetes), several low-abundance taxa exhibited diet-specific patterns. Notably, the mucin-degrading phylum Verrucomicrobia (genus *Akkermansia*) was detected predominantly in the high-protein diets (Groups A and B) and was virtually absent in standard rat feed. This aligns with previous findings that *Akkermansia* genus rise when dietary fiber is limited and protein or chitin is abundant [2]. In contrast, fiber-associated Actinobacteria like *Bifidobacterium* spp. (beneficial carbohydrate fermenters) were present in Group C (standard diet) but did not rise on the *T. molitor* or chicken diets [2]. In fact, no increase in *Bifidobacterium* genus was observed after insect meal supplementation in rats, likely because chitin does not selectively promote this genus. Diet C also harbored unique taxa tied to plant components. For example, the genus *Flavonifractor* which is known to degrade plant flavonoids appeared in standard rat feed group [2], but was nearly absent in Groups A and B, which lack those specific substrates. Conversely, the insect-based diet (Group A) led to the detection of taxa rarely seen in standard rat feed group, such as *Parasporobacterium* sp. and *Turicibacter* sp. These genera remained low in abundance yet were exclusive to Group A, highlighting a niche created by insect-derived nutrients. *Parasporobacterium* sp. (a strictly anaerobic bacterium known to ferment amino acids) and *Turicibacter* sp. have been associated with protein-rich diets in other studies [2]. Their presence in the *T. molitor*-fed rats, even at very low abundance, highlights how adding insect meal introduces specialized microbes adapted to degrade components like chitin and excess peptides that are absent from a grain-based diet.

Zhu et al. [49] analyzed gut microbiota of rats that were fed with either meat proteins (from beef, pork or fish) or non-meat proteins (casein and soy) for 14 days. They showed that the gut bacteria in feces differed depending on the protein content of the diet. Plant protein in the rat diet led to a lower relative abundance of *Lactobacillus* spp. than meat protein. It has been suggested that *Lactobacillus* members play a key role in host metabolic homeostasis, as they can protect the intestinal barrier from damage by pathogens and can reduce inflammation [50]. Consequently, the reduced abundance of *Lactobacillus* spp. in Group A relative to the control groups may represent a concerning indicator.

On the other hand, a beneficial phenomenon may be the increased relative abundance of *Bifidobacterium* spp., *Dubosiella* spp. and *Eubacterium coprostanoligenes* in group A compared to the B and C groups. These bacteria have the ability to reduce cholesterol to coprostanol, but do not require cholesterol for growth [51]. Specific strains of butyrate-producing microbes belonging to the *Eubacterium* genera, may ultimately be considered as beneficial to human health in the same manner as strains of *Lactobacillus* and *Bifidobacterium* species [52]. Our results contradict previous findings, which associated the presence of *Eubacterium* spp. in the gut primarily with increased dietary fiber intake and reported a decrease in their abundance with higher dietary protein and fat content. It should be noted that the studies mentioned did not include insect-derived protein. *Dubosiella* genus is a potential probiotic found in previous research to improve obesity, hypertension and liver disease [53]. Vojinovic et al. [54] demonstrated that *Ruminococcaceae* UCG-005 is positively associated with the metabolism of acetate, the most abundant SCFA produced by bacterial fermentation in the colon. Acetate plays a key role in gut health and host energy metabolism, serving as a major substrate for peripheral tissues, primarily through hepatic metabolism. However, some studies suggest that acetate may mediate the microbiota-brain axis and contribute to the development of metabolic syndrome [54]. Genera from the families Ruminococcaceae and Lachnospiraceae—including *Ruminococcus*, Lachnospiraceae groups NK4A136 and NK4B4, were dominant in group A, and have been reported to participate in the conversion of primary bile acids into secondary bile acids, as well as in the production of SCFAs [54].

The implications of present findings align with a growing body of literature investigating the effects of *T. molitor* meal in other species. The inclusion of *T. molitor* meal has been shown to modulate the gut microbiota in various monogastric species, although the extent and nature of these changes seems to be species dependent. In fish, for instance, the replacement of fishmeal with *T. molitor* meal has typically resulted in only modest alterations in gut microbiota composition. Terova et al. [55] observed that in rainbow trout full replacement of fishmeal with *T. molitor* meal did not significantly influence overall bacterial diversity or richness. Instead, the insect-based diet induced only slight compositional changes, notably a reduction in the relative abundance of certain taxa such as Proteobacteria, and specifically members of the Ruminococcaceae and Neisseriaceae families [55]. These results suggest that the trout gut microbiota exhibited resilience to *T. molitor* meal inclusion, as evidenced by the maintenance of a stable alpha-diversity profile [55]. Nevertheless, other aquaculture studies indicate that insect ingredients can still influence specific microbial groups. For instance, Habte-Tsion et al. [56] reported that partial or complete replacement of fishmeal with defatted *T. molitor* meal in Atlantic salmon significantly altered gut microbiome composition (beta diversity), while alpha-diversity metrics (richness, evenness) remained unaffected. In the aforementioned study, the core microbiota was dominated by *Pseudomonas* spp. in all diets, but fish fed *T. molitor* meal exhibited distinct shifts in community structure and upregulation of immune-related genes (*IgM*, *IgD*, *IgT*), suggesting an enhanced mucosal immune response accompanying the microbiome alterations [56]. Based on presented results and evidence from fish models, it can be concluded that insect meal diets, although not consistently associated with increased abundance of conventionally “beneficial” taxa such as *Lactobacillus* spp., may nonetheless reduce the prevalence of opportunistic or potentially pathogenic microorganisms and modulate host immune responses. These outcomes may be interpreted as indicators of improved gut health, even in the absence of major changes in microbial diversity.

In poultry, the effects of *T. molitor* meal on gut microbiota probably depend on the level of dietary inclusion. Biasato et al. [57] investigated broiler chickens fed incremental levels of yellow mealworm and found significant shifts in gut microbiota composition at higher inclusion percentages. Birds fed 10–15% *T. molitor* meal showed a decrease in the relative abundance of Firmicutes in the cecum and consequently a lower Firmicutes: Bacteroidetes ratio, compared to those on a low 5% inclusion diet [57]. At the genus level, Biasato et al. [57] reported significant increases in *Clostridium* spp., *Alistipes* spp., and *Sutterella* spp. in chickens fed *T. molitor*-supplemented diets, while the beneficial fiber-degrading genus *Ruminococcus* decreased relative to control-fed birds. These changes were dose-dependent and correlated with functional alterations in the gut environment, such as modifications in mucin dynamics. The authors suggested that high dietary inclusion levels (10–15%) of *T. molitor* meal may negatively affect cecal microbial balance or gut mucosal function, while a lower inclusion level (5% *T. molitor*) did not cause such adverse shifts [57], indicating a potential optimal threshold for insect meal inclusion in poultry diets. Overall, the poultry data suggest that moderate inclusion levels of *T. molitor* can be utilized without disrupting the gut ecosystem, whereas excessive levels may disrupt the microbial populations and compromise gut health [58,59].

In swine, studies demonstrate that insect-based diets can beneficially modulate gut microbiota, particularly at moderate inclusion levels. For instance, Yu et al. [60] showed that supplementing finishing pig diets with 4% of black soldier fly *(Hermetia illucens*) larvae meal led to a significant enrichment of beneficial gut bacteria, including *Lactobacillus* spp. as well as SCFAs producers, such as *Pseudobutyrivibrio* spp., *Roseburia* spp., and *Faecalibacterium* spp. In addition, pigs fed a diet containing 4% insect meal exhibited elevated concentrations of total SCFAs, particularly butyrate and isobutyrate, in the colon compared to control animals [60]. This microbial shift was accompanied by reduced concentrations of protein fermentation metabolites (e.g., phenol, p-cresol, etc.), indicating an improved fermentation profile [60]. The overall effect was a favorable gut environment characterized by an anti-inflammatory response, including downregulation of pro-inflammatory mediators (e.g., TLR-4, IFN-γ) and upregulation of anti-inflammatory cytokines (IL-10) and genes associated with barrier function in the insect-fed group [60]. These findings highlight the prebiotic potential of insect components (such as chitin) in swine. Comprehensive analyses of the transcriptome, lipidome and metabolome of key metabolic tissues indicate that partial or complete replacement of a conventional protein source by IM in the diet has only a weak impact on the intermediary metabolism of growing pigs [61]. Meyer et al. [61] concluded that replacement of soybean meal by *T. molitor* larvae meal causes a shift in the cecal microbial community and its fermentation activity in growing pigs. The highest insect meal level resulted in an increase (without statistical significance) in colonic *Lactobacillus* spp. abundance compared to the control diet [29]. This contrasts with the lower doses, where *Lactobacillus* spp. abundance was maintained or increased. These results suggest that, similar to poultry, an optimal inclusion range exists in swine: moderate insect meal supplementation can enhance beneficial taxa and metabolic functions, whereas excessive inclusion might suppress certain beneficial microbes (e.g., lactic acid bacteria) or disrupt the microbiota balance. Overall, swine studies suggests that insect meals, when used in optimal levels, increase SCFA-producing commensals and reduce potential pathogens, which can translate into improved gut health and immunity in pigs [31,60,61].

Compared to studies in livestock, research using rodent models to investigate the effects of insect meal remains relatively limited. Nevertheless, recent evidence suggests that rodents exhibit generally similar microbiota modulation responses to edible insects. Saeb et al. [62] demonstrated that feeding purified *T. molitor* cuticles (a chitin-rich fraction) to obese rats favorably reshaped their gut microbiome. Specifically, the study reported an increase in the abundance of certain beneficial bacterial families and an overall shift in community structure that was deemed advantageous for host health [61]. These microbiota changes were accompanied by metabolic improvements—notably attenuation of high-fat-diet-induced hepatic steatosis in the obese rat model—underscoring a functional benefit of insect-derived prebiotic fibers. While direct studies in healthy rodents are limited, the results by Saeb et al. [62] support the hypothesis that chitin and other bioactive *T. molitor*-derived components may function as prebiotic substrates, promoting the growth of gut bacteria that confer health benefits to the host. These taxonomic shifts differ from those observed with the whole-meal diet, which did not increase bifidobacteria or lactobacilli, suggesting that the chitin-rich fraction selectively enriches carbohydrate—and mucus-utilizing taxa, whereas the high-protein/full-fat meal favors proteolytic Firmicutes [62]. In other studies, BALB/c mice fed a diet containing 20% exuviae for eight weeks exhibited significant enrichment of Bifidobacteriaceae and Lactobacillaceae, along with a modest increase in lactic-acid bacteria counts, with no reported loss of diversity [63]. Again, the fiber-rich exuviae promoted classic probiotic families, contrasting with our protein-dominated formulation where lactobacilli were slightly lower than controls. These discrepancies show the importance of the insect fraction used (protein vs. lipid vs. chitinous cuticle) in steering microbial responses. Kang et al. [64] stated that replacing 50–100% of dietary protein with defatted *T. molitor* meal for eight weeks increased fecal and cecal alpha-diversity and influenced >45 genera, producing a clear treatment-specific β-diversity separation. Although detailed genus-level shifts differed from those observed in our healthy-rat model, both studies demonstrate that high levels of *T. molitor* inclusions remodel gut microbiota composition without inducing dysbiosis; rather, community richness is either maintained (present study) or enhanced [64].

Overall, the cross-species evidence aligns with the outcomes of our rat study, suggesting that insect-based nutrition generally promotes a more diverse and metabolically active gut microbiota without inducing dysbiosis or functional impairment. While small differences exist, fish and pigs often exhibit increased microbial diversity with insect diets, whereas poultry and rodents may require moderate inclusion levels to prevent minor imbalances, but the trend is that gut homeostasis is maintained or improved with *T. molitor* supplementation [65,66]. This resilience of the gut microbiome, even when exposed to novel dietary ingredients such as insects, is encouraging for the future of sustainable feeds. The fact that beneficial genera (e.g., *Bifidobacterium*, *Ruminococcus*) and microbial metabolites frequently increase is particularly promising, as these are linked to enhanced nutrient absorption, gut barrier integrity, and anti-inflammatory effects across hosts. Preliminary human trials have similarly shown that consuming edible insects (*Acheta domesticus*) can increase gut probiotic bacteria and reduce inflammatory markers [67], suggesting that the microbiota-mediated benefits observed in animals may translate to humans. Moving forward, integrating metagenomic and metatranscriptomic analyses [68,69,70] will be crucial to elucidate the specific functional pathways modulated by insect-based diets (e.g., bile acid metabolism, vitamin synthesis).

A final point worth considering is that the *T. molitor* itself is not microbiologically neutral: several studies showed that *T. molitor* carries a characteristic core microbial population dominated by spore-forming Firmicutes (e.g., *Bacillus* spp., *Enterococcus* spp.) and a smaller set of Proteobacteria that may survive mild processing and remain detectable in finished ingredients. High-throughput sequencing of commercial powders and snacks revealed that products cluster by insect species, with only a handful of highly prevalent taxa forming a *T. molitor*-specific microbial signature that persists despite heating, grinding, or mixing [21,22,71]. A culture-based survey recorded total viable counts around 10^5^–10^8^ CFU/g, largely attributable to *Bacillus cereus*-group spores and lactic cocci [72]. Although a brief starvation–heat treatment can eradicate vegetative cells, resilient spores may remain and potentially colonize the consumer’s gut. The larval gut itself is similarly enriched in Firmicutes: in the comprehensive metagenomic profile assembled by Khanal et al. [73], Firmicutes (mainly *Bacillus* spp., *Lysinibacillus* spp. and *Enterococcus* spp.) accounted for over 70% of all reads, with Proteobacteria comprising most of the remainder. Because many of these genera harbor chitinases and SCFAs biosynthetic pathways, this might explain the rise in *Dubosiella* spp., *Eubacterium coprostanoligenes* group observed in *T. molitor*-fed rats. Taken together, these lines of evidence indicate that part of the microbiota shift seen after high-level *T. molitor* inclusion may derive from insect-associated bacteria and spores acting as colonizers or metabolic catalysts once inside the vertebrate gut. Continual screening of *T. molitor* microbiota, both for beneficial strains and for potential pathogens, therefore, remains critical when formulating next-generation feeds.

This study has certain limitations. Metagenomic analyses used two pooled fecal replicates per diet group, each representing all 15 animals, which reduces the number of statistical units and therefore limits power for between-group hypothesis tests. As a result, some contrasts did not reach *p* < 0.05 despite large effect sizes (e.g., PERMANOVA R^2^ up to 0.86) and consistent diet-specific clustering in ordination plots. While deep sequencing and concordant results across multiple analyses strengthen the robustness of the observed trends, findings should be interpreted with emphasis on effect sizes and directionality rather than on *p*-values alone. Notably, several culture-based endpoints were significant, supporting biological relevance; however, future studies with additional biological replicates and/or individual sampling will be needed to increase statistical power and enable more detailed inferences.

## 4. Materials and Methods

### 4.1. Feed Preparation

A feed formulation meeting the nutritional guidelines of the European Pet Food Industry Federation (FEDIAF) was developed. Live *T. molitor* larvae were sourced from a national insect farm (Tenebria, Lubawa, Poland) certified under a Hazard Analysis and Critical Control Points (HACCP) system. The farm operates in compliance with Polish veterinary regulations concerning food safety of animal origin (Official Journal of Laws of the Republic of Poland 2019, item 1252), animal health and infectious disease control (Official Journal of Laws of the Republic of Poland 2020, items 148, 285), animal welfare (Official Journal of Laws of the Republic of Poland 2019, items 122, 1123), and feed (Official Journal of Laws of the Republic of Poland 2019, item 269).

Prior to processing, *T. molitor* larvae were fasted for 72 h to clear their digestive tracts of any residual feed. The insects also underwent mechanical cleaning to remove remnants of feed, excreta, molted exoskeletons, and any dead or pupated individuals. Euthanasia was performed by thermal methods in accordance with the American Veterinary Medical Association (AVMA) Guidelines for the Euthanasia of Animals. A standardized meal was then produced using a dedicated technological line designed for insect killing and comprehensive processing. The Quality Management System implemented during feed production was compliant with both the international ISO 9001 standard [74] and HACCP principles. The final feed was produced in a dry form, shaped into oval granules with a diameter of approximately 15 mm. Two batches were prepared: insect-based feed- containing 35% full-fat, non-dechitinized *T. molitor* meal; poultry-based feed: containing 35% commercially available poultry meal. All other feed components remained identical between the two batches, and the complete formulation is covered under a patent application P.443579. These formulations were intended to create a hypoallergenic feed for dogs suffering from food-responsive enteropathies [75]. A detailed description of the feed was presented in the article by Gałęcki et al. [75].

### 4.2. Laboratory Animals and Experimental Procedure

This study was conducted following the 3R principle (Replacement, Reduction, and Refinement) [76,77]. The experimental model consisted of Wistar rats (Rattus norvegicus), selected for their suitability in physiological and nutritional research relevant to human gastrointestinal studies. Female rats, 12 months old and weighing 250–280 g from the same breeding facility, were acclimatized for 14 days prior to the experiment. During this quarantine period, they were housed under controlled environmental conditions: a minimum floor area of 800 cm^2^ per cage, a minimum cage height of 18 cm, temperature maintained at 22 ± 2 °C, relative humidity at 60 ± 5%, and an artificial light cycle of 12 h per day at an intensity of 65 lux. All animals were housed in a specialized animal facility designed to meet laboratory standards. Three experimental groups were formed, each consisting of 15 rats in individually housed cages: Group A (Experimental)—feed containing 35% *T. molitor* meal; Group B (Positive Control)—feed containing 35% poultry meal; Group C (Negative Control)—a standard laboratory rat feed (Labofeed H Standard, Wytwórnia Pasz “Morawski,” Żurawia, Poland). All rats were maintained on their respective diets for 9 weeks. The daily feed ration was adjusted to meet the energy requirements of the animals, and water was provided ad libitum. The study adhered to the ARRIVE guidelines for reporting animal research. All procedures were approved by the Local Ethics Committee (decision no. 31/2022 of 18 May 2020) and conducted in accordance with Polish legislation (Act of 15 January 2015, on the protection of animals used for scientific or educational purposes, Official Journal of 2015, item 266) and Directive 2010/63/EU of the European Parliament and of the Council of 22 September 2010, on the protection of animals used for scientific purposes.

### 4.3. Microbiological Analysis of Rats Feces

Fecal samples were collected after 4, 6, and 8 weeks of feeding (Appendix A). Freshly excreted fecal pellets were immediately placed in sterile tubes and stored at 4 °C until analysis. A 1 g portion of feces was transferred into a sterile homogenization bag containing 99 mL of sterile 0.85% NaCl solution, then homogenized and analyzed without delay. The homogenates were serially diluted (from 10^−2^ to 10^−8^) in triplicate and plated on appropriate selective culture media and incubated. Selective media and incubation conditions used in the research are shown in Supplementary Materials, Table S2. After incubation, colonies representing each targeted microbial group were counted, and results were expressed as colony-forming units per gram of feces (CFU/g). For each microorganism in total, n = 405 cultures were performed, 135 for each group.

### 4.4. Statistical Analysis of Microbiological Counts

Prior to parametric testing, the assumptions of normality and homogeneity of variances were examined. Normality of residuals was evaluated with the Shapiro–Wilk test, and homogeneity of variances with Levene’s test. Both assumptions were met (*p* > 0.05). Statistically significant differences in bacterial counts were assessed using analysis of variance (ANOVA), with group as the independent factor and time (weeks 4, 6, and 8), including their interaction. When the ANOVA indicated significant effects, Tukey’s honestly significant difference (HSD) test was applied for post hoc pairwise comparisons. Statistical significance was set at *p* < 0.05. Results are presented as mean ± SD and on the log_10_ scale where appropriate. All calculations were performed in Statistica 13.3 (TIBCO Software Inc., Palo Alto, CA, USA).

### 4.5. Microbiological Biodiversity of Rat Feces on the Basis of High-Throughput Sequencing

Fecal samples were collected 9 weeks following the initiation of the experiment. Feces were taken from the rectum during necropsy of the animals. Immediately after collection, fecal samples were frozen and stored at −80 °C until further processing, after which genomic DNA was extracted from two replicates of pooled fecal samples (n = 15 for each pooled samples per group) for groups A, B, and C using a commercial kit (Genomic Mini AX Stool, A&A Biotechnology, Gdańsk, Poland) following the manufacturer’s instructions; in this study, samples labeled A1 and A2 represented rats fed a 35% *T. molitor* meal (Group A), B1 and B2 represented rats fed a 35% chicken meal (Group B), and C1 and C2 corresponded to animals receiving a commercial standard rat feed. DNA concentration and purity were assessed via NanoDrop spectrophotometry (Thermo Fisher Scientific, Waltham, MA, USA), with purity judged by the A260/A280 ratio. The extracted DNA was stored at −20 °C prior to metagenomic analysis by next-generation sequencing (NGS).

Metagenomic analysis of bacterial and archaeal communities focused on the hypervariable V3–V4 region of the 16S rRNA gene. Amplification of this region and library preparation were carried out using the specific primer sets 341F and 785R (16S-based analysis) together with Q5 Hot Start High-Fidelity 2X Master Mix (New England Biolabs, Ipswich, MA, USA), following the manufacturer’s recommended protocol. Sequencing was performed on a MiSeq platform (Illumina, San Diego, CA, USA) using a paired-end (PE) 2 × 300 nt run with the Illumina v3 kit. Initial data processing (demultiplexing and preliminary quality control) was conducted automatically on the MiSeq system using MiSeq Reporter (MSR) v2.6.

Bioinformatic analysis, which classified the reads down to the species level, was performed using QIIME 2 in conjunction with the Silva 138 reference sequence database. The Divisive Amplicon Denoising Algorithm 2 (DADA2) pipeline was used to remove sequencing artifacts and generate Amplicon Sequence Variants (ASVs). Quality control steps included error profile analysis, dynamic quality filtering, removal of adapter sequences, and discarding reads shorter than 30 nucleotides. Denoising, merging paired-end reads, dereplication, and chimera filtering were also performed. ASVs (or OTUs) were clustered at a similarity threshold of ≥97%. Taxonomic assignment of the ASVs/OTUs was based on the Silva 138 database. All sequencing data were analyzed in R software version 3.5.1. Alpha diversity metrics (ACE, Chao1, Observed Species, Shannon, Simpson and inverted Simpson indices) were calculated using QIIME 2 to assess within-sample diversity. Beta diversity was examined using Bray–Curtis distances and interpreted via Principal Component Analysis (PCA) and Principal Coordinates Analysis (PCoA). Plots were generated with the ggplot2 (version 3.0.0) package in R. Predicted functional shifts in the gut microbiome of rats were based on Phylogenetic Investigation of Communities by Reconstruction of Unobserved States (PICRUSt2 version 2.6.2). For the purposes of this article, the results for individual groups were averaged. Sequencing data files in the FASTQ format were deposited in the NCBI Sequence Read Archive (SRA) under BioProject accession number PRJNA1019627 (BioSample Acc. SAMN37487378—SAMN37487383).

## 5. Conclusions

This study provides comprehensive microbiological and metagenomic evidence that incorporating *T. molitor* meal at 35% dietary inclusion level in laboratory rat feed does not adversely affect gut microbiota composition or diversity. Instead, it induces specific, functionally relevant shifts in the microbial community structure. Compared to rats fed standard or chicken-based diets, the *T. molitor*-fed group demonstrated significantly lower counts of total aerobic and anaerobic bacteria, fungi, and coagulase-positive staphylococci—suggesting a potential modulatory or even protective effect of *T. molitor*-based diets on gut microbial load. Metagenomic analysis further revealed that the *T. molitor* diet enriched specific bacterial clades associated with amino acid metabolism, cholesterol reduction, and SCFAs production, including members of the Oscillospirales, Ruminococcaceae, and *Eubacterium coprostanoligenes* group. These shifts were accompanied by a reduction in mucin-degrading genera such as *Akkermansia* spp. and fermentative Bacteroidetes like Muribaculaceae, indicating a microbial profile adapted to high-protein, low-fiber substrates. Functional predictions confirmed the dual upregulation of protein fermentation and complex glycan degradation pathways, consistent with the biochemical composition of *T. molitor* meal. These observations highlight the resilience of the gut microbiome to dietary innovation and support the potential of *T. molitor* as a sustainable, functional protein source. Taken together, these results suggest that *T. molitor* meal is a viable component of high-protein diets, capable of supporting a balanced gut microbial ecosystem. Its inclusion may also provide benefits such as enhanced gut fermentation capacity and microbial metabolism without inducing dysbiosis. Future studies should investigate the long-term physiological effects of such dietary interventions, including impacts on systemic immunity, metabolic health, and performance parameters across various animal models. As the demand for sustainable protein sources continues to rise, *T. molitor* represents a promising candidate for promoting intestinal microbial homeostasis across species. An innovative aspect of this study is the indication that *T. molitor* meal may act not only as a nutrient source but also as a microbiome modulator, potentially delivering insect-associated microbial strains and metabolites that synergistically enhance host gut function. This dual role, both feed and microbial ‘biostimulant’, opens new possibilities for designing insect-based diets that harness beneficial microbe–host interactions, contributing to improved animal health and resilience. Future research integrating multi-omics approaches could unravel these complex dynamics, creating the way for precision nutrition strategies using edible insects.

## Data Availability

The original data presented in the study are openly available The National Center for Biotechnology Information Sequence Read Archive (SRA) under BioProject accession number PRJNA1019627 (BioSample Acc. SAMN37487378—SAMN37487383).

## Supplementary Materials

The following supporting information can be downloaded at https://www.mdpi.com/article/10.3390/ijms26178663/s1.

## Appendix A

Baseline (day 0) fecal sampling was omitted for both ethical and methodological reasons. All animals originated from the same breeding colony, underwent a uniform 14-day acclimatization period, and consumed the identical standard diet that ultimately served as the negative-control ration for Group C. Consequently, inter-individual variability of the gut microbiota at the start of the feeding trial was expected to be negligible, making any statistically significant differences between groups highly improbable. From the standpoint of the 3R framework, collecting needless baseline material would have (i) increased animal handling and attendant stress, thereby violating Refinement, and (ii) consumed additional culture media, disposables, energy, and labor, conflicting with Reduction by enlarging experimental resource use without an accompanying scientific benefit. Moreover, handling-induced stress is known to transiently alter gastrointestinal transit and enteric microbial composition; an extra sampling point could therefore have introduced a confound rather than a useful covariate. Finally, because the entire population was kept in identical cages within a single dedicated room, the risk of cage- or room-level microbiota drift on day 0 was effectively zero. For these reasons, and in alignment with local ethics-committee guidance that discourages procedures without clear scientific justification, the authors judged day 0 fecal sampling to be both economically and scientifically unwarranted and therefore omitted it from the design.
