# Supplementary material for: Tenebrio molitor Meal-Induced Changes in Rat Gut Microbiota: Microbiological and Metagenomic Findings
Source: Int J Mol Sci. 2025 Sep 5;26(17):8663. doi: 10.3390/ijms26178663 (PMC12428859; doi:10.3390/ijms26178663)
Supplement: Supplementary file 1 [file ijms-26-08663-s001.zip › Figure S1.pdf]

International Journal of
Molecular Sciences

Figure S1. Heat tree visualization for group: A (n=15, 35% *T. molitor* meal), B (n=15, 35% chicken meal), and C (n=15, standard rat feed).

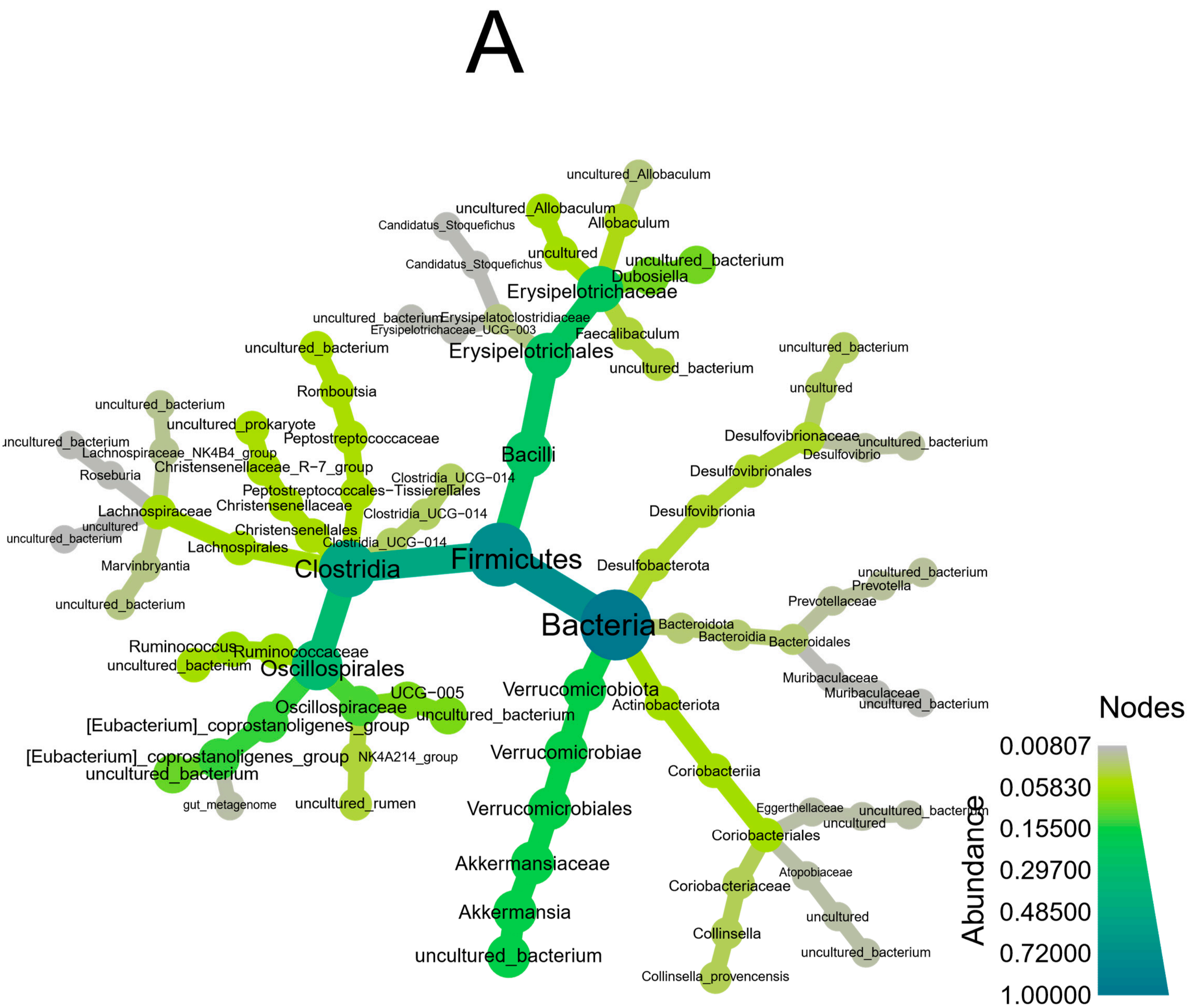

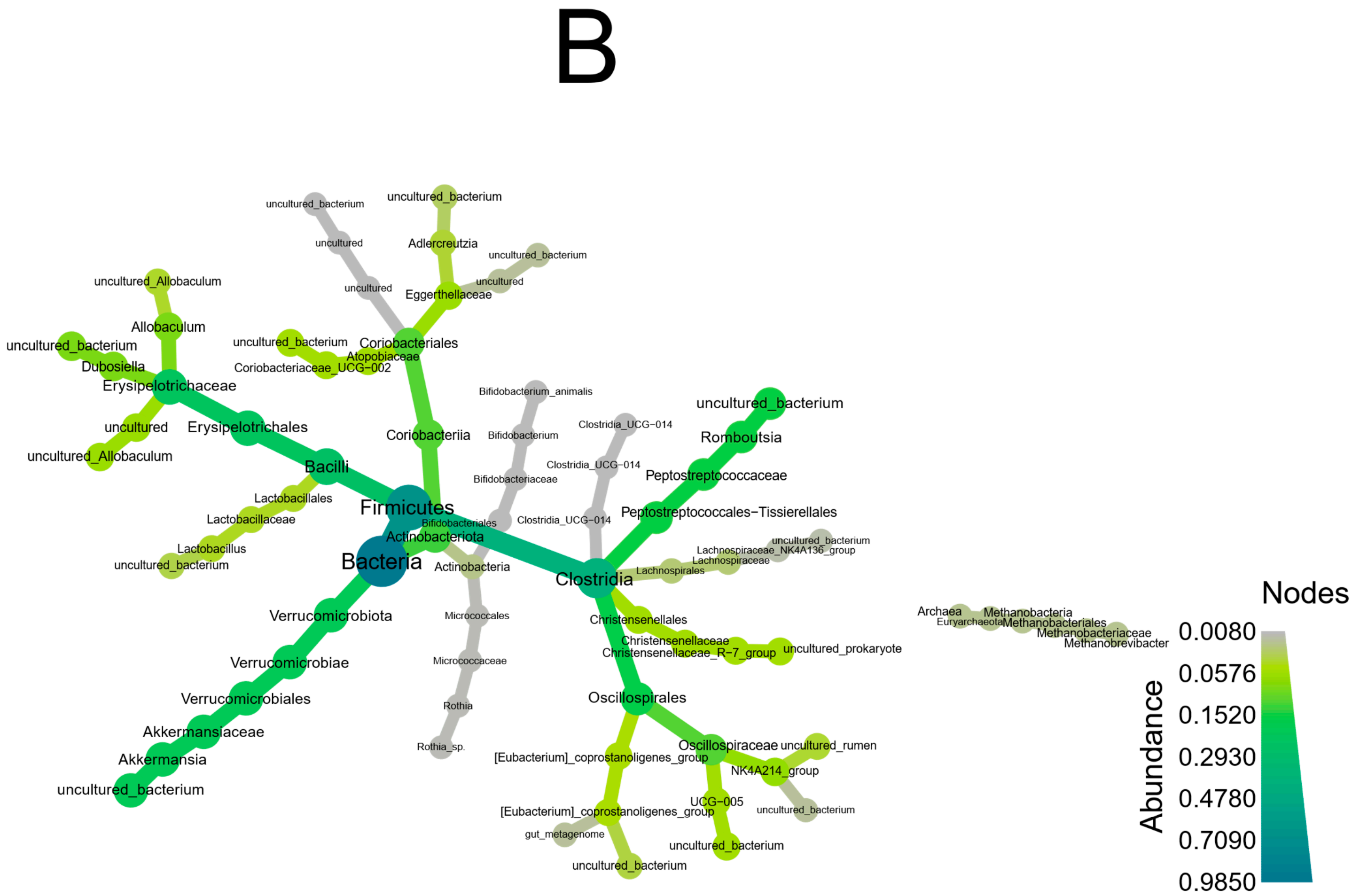
B
Bacteria
Firmicutes
Actinobacteriota
Verrucomicrobiota
Verrucomicrobiae
Verrucomicrobiales
Akkermansiaceae
Akkermansia
uncultured_bacterium
Bacilli
Erysipelotrichales
Erysipelotrichaceae
Allobaculum
uncultured_Allobaculum
uncultured_bacterium
Dubosiella
uncultured
uncultured_Allobaculum
Lactobacillales
Lactobacillaceae
Lactobacillus
uncultured_bacterium
Coriobacteriia
Coriobacteriales
Atopobiaceae
Coriobacteriaceae_UCG−002
uncultured_bacterium
Eggerthellaceae
Adlercreutzia
uncultured_bacterium
uncultured
uncultured_bacterium
uncultured
uncultured
uncultured_bacterium
Actinobacteria
Bifidobacteriales
Bifidobacteriaceae
Bifidobacterium
Bifidobacterium_animalis
Micrococcales
Micrococcaceae
Rothia
Rothia_sp.
Clostridia
Clostridia_UCG−014
Clostridia_UCG−014
Clostridia_UCG−014
Peptostreptococcales−Tissierellales
Peptostreptococcaceae
Romboutsia
uncultured_bacterium
Lachnospirales
Lachnospiraceae
Lachnospiraceae_NK4A136_group
uncultured_bacterium
Christensenellales
Christensenellaceae
Christensenellaceae_R−7_group
uncultured_prokaryote
Oscillospirales
Oscillospiraceae
uncultured_rumen
NK4A214_group
uncultured_bacterium
UCG−005
uncultured_bacterium
[Eubacterium]_coprostanoligenes_group
[Eubacterium]_coprostanoligenes_group
gut_metagenome
uncultured_bacterium
Archaea
Euryarchaeota
Methanobacteria
Methanobacteriales
Methanobacteriaceae
Methanobrevibacter
Nodes
Abundance
0.0080
0.0576
0.1520
0.2930
0.4780
0.7090
0.9850

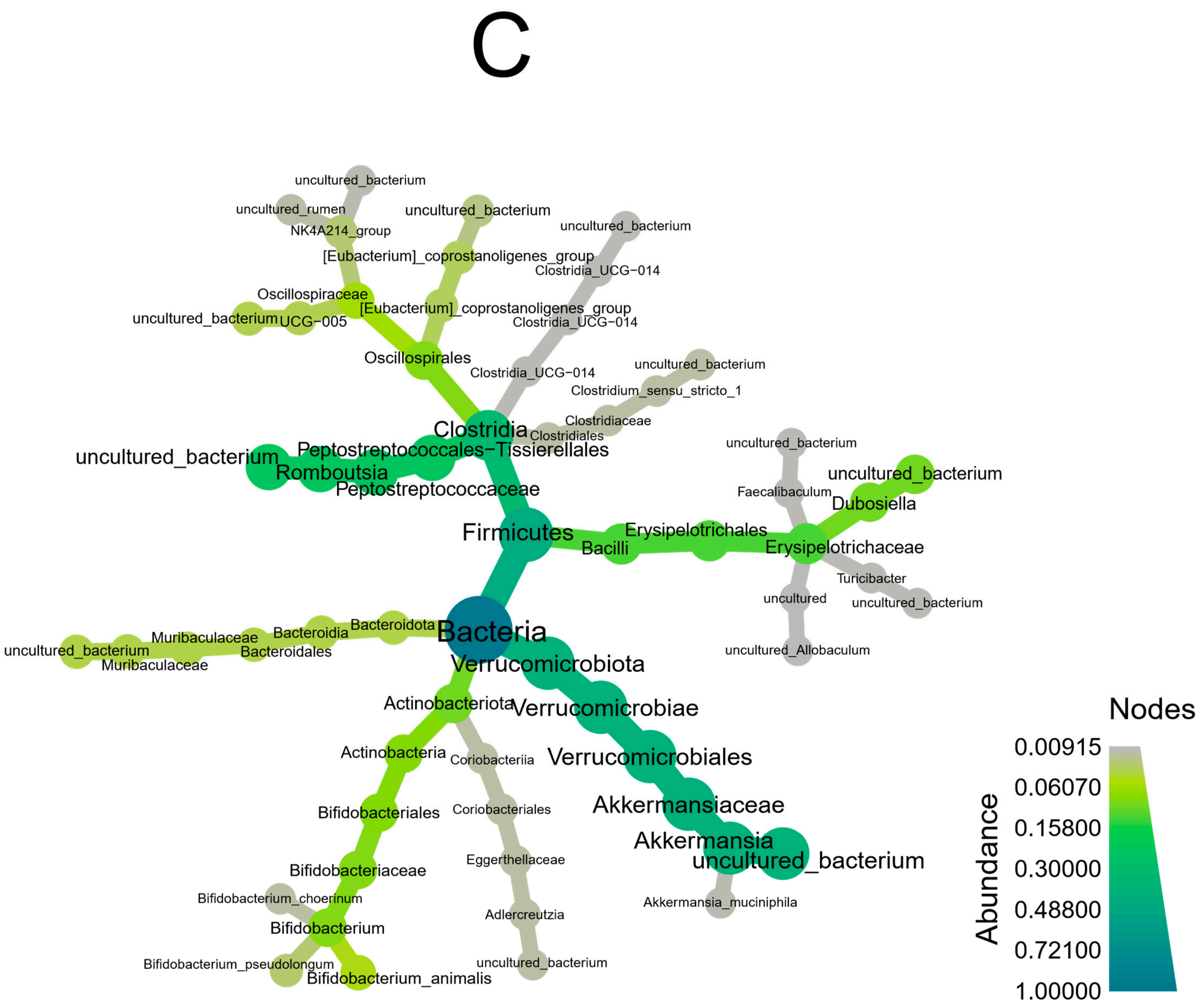
C
Bacteria
Firmicutes
Clostridia
Bacilli
Erysipelotrichales
Erysipelotrichaceae
Dubosiella
uncultured_bacterium
Faecalibaculum
Turicibacter
uncultured
uncultured_Allobaculum
Peptostreptococcales−Tissierellales
Peptostreptococcaceae
Romboutsia
Oscillospirales
Oscillospiraceae
UCG−005
NK4A214_group
uncultured_rumen
[Eubacterium]_coprostanoligenes_group
Clostridia_UCG−014
Clostridium_sensu_stricto_1
Clostridiaceae
Clostridiales
Bacteroidota
Bacteroidia
Bacteroidales
Muribaculaceae
Verrucomicrobiota
Verrucomicrobiae
Verrucomicrobiales
Akkermansiaceae
Akkermansia
Akkermansia_muciniphila
Actinobacteriota
Actinobacteria
Bifidobacteriales
Bifidobacteriaceae
Bifidobacterium
Bifidobacterium_choerinum
Bifidobacterium_pseudolongum
Bifidobacterium_animalis
Coriobacteriia
Coriobacteriales
Eggerthellaceae
Adlercreutzia
Nodes
Abundance
0.00915
0.06070
0.15800
0.30000
0.48800
0.72100
1.00000
