# Supplementary material for: Tenebrio molitor Meal-Induced Changes in Rat Gut Microbiota: Microbiological and Metagenomic Findings
Source: Int J Mol Sci. 2025 Sep 5;26(17):8663. doi: 10.3390/ijms26178663 (PMC12428859; doi:10.3390/ijms26178663)
Supplement: Supplementary file 1 [file ijms-26-08663-s001.zip › Table S1.pdf]

International Journal of
Molecular Sciences

Table S1. Average microbial counts (mean ±SD) in rat feces across study groups at weeks 4, 6, and 8.

Group	Number of microorganisms, CFU/g								
	A			B			C		
Week	4	6	8	4	6	8	4	6	8
Aerobic bacteria	2.49x10^{7}	1.38x10^{7}	3.90x10^{7}	7.40x10^{7}	5.64x10^{7}	6.52x10^{7}	1.93x10^{8}	2.19x10^{8}	2.35x10^{8}
	±2.19x10^{7}	±1.86x10^{7}	±1.83x10^{7}	±8.39x10^{7}	±7.10x10^{7}	±5.26x10^{7}	±1.10x10^{8}	±1.22x10^{8}	±2.83x10^{8}
Clostridium spp.	1.36x10^{3}	1.19x10^{3}	1.08x10^{3}	9.96x10^{2}	1.16x10^{3}	1.10x10^{3}	1.55x10^{3}	1.71x10^{3}	1.42x10^{3}
	±1.45x10^{3}	±1.39x10^{3}	±6.59x10^{2}	±5.56x10^{2}	±7.31x10^{2}	±6.86x10^{2}	±2.45x10^{3}	±3.15x10^{3}	±2.07x10^{3}
Lactobacillus spp.	1.15x10^{8}	1.31x10^{8}	1.40x10^{8}	2.65x10^{8}	2.31x10^{8}	2.42x10^{8}	3.58x10^{8}	3.89x10^{8}	3.75x10^{8}
	±7.19x10^{7}	±1.38x10^{8}	±1.02x10^{8}	±2.56x10^{8}	±4.54x10^{8}	±2.96x10^{8}	±1.67x10^{8}	±3.45x10^{8}	±1.48x10^{8}
Enterococcus spp.	1.84x10^{7}	2.18x10^{7}	2.16x10^{7}	1.23x10^{7}	1.03x10^{7}	1.40x10^{7}	2.34x10^{7}	2.00x10^{7}	2.10x10^{7}
	±6.25x10^{6}	±2.42x10^{7}	±5.28x10^{7}	±2.39x10^{6}	±1.32x10^{7}	±2.56x10^{7}	±2.53x10^{7}	±3.12x10^{7}	±1.57x10^{7}
Bacteroides spp.	2.60x10^{8}	3.02x10^{8}	3.51x10^{8}	3.75x10^{8}	3.52x10^{8}	2.76x10^{8}	6.41x10^{8}	6.35x10^{8}	6.52x10^{8}
	±2.03x10^{8}	±2.02x10^{8}	±2.58x10^{8}	±2.15x10^{8}	±4.42x10^{8}	±2.19x10^{8}	±9.97x10^{8}	±3.92x10^{8}	±6.59x10^{8}
Enterobacteriaceae	3.32x10^{5}	3.14x10^{5}	2.98x10^{5}	3.90x10^{5}	3.81x10^{5}	4.41x10^{5}	3.99x10^{5}	3.90x10^{5}	4.18x10^{5}
	±3.28x10^{5}	±2.71x10^{5}	±1.91x10^{5}	±2.85x10^{5}	±2.48x10^{5}	±9.95x10^{5}	±6.21x10^{5}	±3.16x10^{5}	±2.99x10^{5}
Staphylococcus spp.	1.37x10^{5}	1.90x10^{5}	4.74x10^{5}	3.85x10^{5}	4.22x10^{5}	4.19x10^{5}	2.62x10^{5}	2.92x10^{5}	3.42x10^{5}
	±1.41x10^{5}	±1.89x10^{5}	±1.23x10^{6}	±4.22x10^{5}	±3.93x10^{5}	±1.74x10^{5}	±2.30x10^{5}	±3.29x10^{5}	±1.99x10^{5}
Anaerobic bacteria	2.09x10^{8}	3.28x10^{8}	2.70x10^{8}	5.44x10^{8}	5.74x10^{8}	5.91x10^{8}	4.82x10^{8}	4.91x10^{8}	4.95x10^{8}
	±1.23x10^{8}	±1.92x10^{8}	±2.02x10^{8}	±1.73x10^{8}	±4.17x10	±7.11x10^{8}	±3.90x10^{8}	±3.11x10^{8}	±4.70x10^{8}
Fungi	1.71x10^{3}	5.00x10^{3}	3.11x10^{3}	3.15x10^{3}	4.74x10^{3}	4.87x10^{3}	3.45x10^{3}	4.83x10^{3}	5.95x10^{3}
	±1.27x10^{3}	±3.21x10^{3}	±5.66x10^{3}	±6.33x10^{3}	±4.19x10^{7}	±7.59x10^{3}	±4.42x10^{3}	±3.40x10^{2}	±6.00x10^{3}
