# Supplementary material for: Tenebrio molitor Meal-Induced Changes in Rat Gut Microbiota: Microbiological and Metagenomic Findings
Source: Int J Mol Sci. 2025 Sep 5;26(17):8663. doi: 10.3390/ijms26178663 (PMC12428859; doi:10.3390/ijms26178663)
Supplement: Supplementary file 1 [file ijms-26-08663-s001.zip › Table S2.pdf]

International Journal of Molecular Sciences

Table S2. Media used in microbial analysis.

Medium	Supplier	Microorganisms	Incubation conditions
De Man, Rogosa and Sharpe with agar (MRS Agar) with 0.2% nystatin	Merck, Germany	*Lactobacillus* spp.	37°C ± 2°C 24–48 h*
Violet Red Bile Glucose Agar (VRBG) with 0.2% nystatin	Merck, Germany	Enterobacteriaceae	37°C ± 2°C 24–48 h
Tryptose sulfite cycloserine agar (TSC Agar), with 0.2% nystatin and 0.04% cycloserine	Merck, Germany	*Clostridium* spp.	37°C ± 2°C 24–48 h*
Bile Aesculin Agar (BAA Agar)	Merck, Germany	*Enterococcus* spp.	37°C ± 2°C 24–48 h
Schaedler Agar	Merck, Germany	*Bacteroides* spp.	37°C ± 2°C 24–48 h*
Baird Parker Agar with 0.2% nystatin	Merck, Germany	Coagulase positive *Staphylococcus* spp.	37°C ± 2°C 24–48 h
Tryptic Soy Agar (TSA) with 0.2% nystatin	Merck, Germany	Total number of bacteria	30°C ± 2°C 48 h
Brain Heart Infusion Agar (BHI Agar)	Merck, Germany	Total numer of anaerobic bacteria	37°C ± 2°C 24–48 h*
Malt Extract Agar (MEA) with 0.1% chloramphenicol	Merck, Germany	Total number of fungi (yeast and moulds)	25°C ± 2°C 7 days

* - anaerobic conditions
